# Priming VRC01-precursor B cells with non-envelope immunogens disfavors boosting with HIV-1 envelope

**DOI:** 10.1038/s41541-025-01235-5

**Published:** 2025-08-05

**Authors:** Andrew Wilcox-King, Yu-Hsin Wan, Samuel C. Scharffenberger, Crystal B. Chhan, Amelia R. Davis, Leah J. Homad, Emilie Seydoux, Kellie J. MacPhee, Latha Kallur Siddaramaiah, Mariane Melo, Pia Dosenovic, Darrell J. Irvine, Ollivier Hyrien, Leonidas Stamatatos, Andrew T. McGuire

**Affiliations:** 1 Vaccine and Infectious Disease Division, Fred Hutchinson Cancer Center, Seattle, WA USA; 2 Koch Institute for Integrative Cancer Research, Massachusetts Institute of Technology, Cambridge, MA USA; 3 Howard Hughes Medical Institute, Chevy Chase, MD USA; 4 Department of Microbiology, Tumor and Cell Biology, Division of Virology and Immunology, Karolinska Institutet, Solna, Sweden; 5 Ragon Institute of Massachusetts General Hospital, Massachusetts Institute of Technology, and Harvard University, Cambridge, MA USA; 6 Harvard-MIT Health Sciences and Technology Program, Institute for Medical Engineering and Science, Massachusetts Institute of Technology, Cambridge, MA USA; 7 Departments of Biological Engineering and Materials Science and Engineering, Massachusetts Institute of Technology, Cambridge, MA USA; 8 Department of Global Health, University of Washington, Seattle, WA USA; 9 Department of Laboratory Medicine and Pathology, University of Washington, Seattle, WA USA

**Keywords:** HIV infections, Immunology, Protein vaccines

## Abstract

VRC01-class antibodies are a genetically restricted class of antibodies capable of potently neutralizing diverse strains of HIV-1. Unmutated VRC01 precursors fail to recognize recombinant HIV-1 Envelope (Env) proteins, which necessitated the development of germline targeting vaccine immunogens capable of initiating VRC01-class B cell response. Among these, we developed an anti-idiotypic monoclonal antibody (ai-mAb)-derived VRC01 class targeting immunogen. Because it is distinct from Env, we speculated that the ai-mAb will selectively engage naive VRC01 class B cells while limiting B cell responses directed at off-target epitopes on Env during prime-boost regimens. Here, we evaluated the serum and B cell responses to ai-mAb prime/Env boost, and Env-prime/Env boost regimens in a murine adoptive transfer model where VRC01 precursor B cells are present at physiological levels. We found that the Env-Env regimen led to the greatest expansion of on-target VRC01 B cells, drove larger VRC01-class GC responses, and elicited higher titers of circulating antibodies despite also eliciting substantial off-target Env-specific responses. Single-cell sorting experiments revealed that the ai-mAb was driving off-track somatic mutations. IgG transfer experiments demonstrated that circulating off-target antibodies provide a positive feedback mechanism that potentiates on-target B cell responses. Collectively, the results suggest that non-Env immunogens are not ideal for priming VRC01-class B cells, where sequential boosting with Env will be required to drive maturation of neutralizing breadth.

## Introduction

Numerous monoclonal antibodies have been isolated from HIV-1-infected individuals that are capable of potently neutralizing diverse strains of HIV-1^1–3^. These have been termed broadly neutralizing antibodies (bNAbs). Among these, VRC01-class antibodies have been isolated from several different donors and all have independently arisen from B cells expressing B cell receptors (BCRs) derived from the VH1-2∗02 variable heavy (VH) gene, paired with light chains (LCs) expressing rare 5-aa CDRL3 domains^4–15^. VRC01 class antibodies are up to ∼40% mutated from their chromosomally encoded (germline) sequence and diverge in sequence from one another, yet bind the CD4 binding site (CD4-BS) on Envelope (Env) in a nearly identical complementary determining heavy chain region 2 (CDHR2)-dominated manner^6–9,11,12,16,17^.

When passively delivered, or genetically expressed in animal models, VRC01-class monoclonal antibodies (mAbs) confer protection from experimental HIV-1 infection, indicating that if elicited by vaccination, they could be protective against susceptible viral strains in humans^18–22^. In support of this, passive delivery of VRC01 protected high-risk individuals from acquisition of susceptible HIV-1 strains^23^. These findings suggest that eliciting VRC01-class antibodies will be an important goal of an effective HIV-1 vaccine.

It is possible to generate inferred germline (iGL) versions of VRC01-class mAbs isolated from HIV-1-infected donors through homology-guided reversion of antibody variable (V) and joining (J) gene-encoded portions of the mutated heavy chains (HCs) to their chromosomally templated sequences; however, these iGL mAbs fail to bind recombinant Env and they do not neutralize HIV-1^4,7,24–27^. These findings motivated the development of germline-targeting immunogens that can engage unmutated VRC01 precursor B cells^25,26,28–30^, some of which are currently being evaluated in clinical trials (^31^ and NCT05471076, NCT04224701, NCT03547245, NCT06006546).

We developed anti-idiotypic monoclonal antibodies (ai-mAbs) as a strategy to target unmutated BCRs with genetic features associated with bNAbs^32–34^. We previously engineered a bispecific anti-idiotypic molecule in which one arm is derived from an ai-mAb, called iv9, that preferentially recognizes all allelic variants of VH1-2 HCs, and a second arm derived from an ai-mAb, called iv4, that preferentially recognizes LCs with 5-aa CDRL3^34^. The bispecific ai-mAb, known as iv4/iv9, specifically activated iGL-VRC01 B cells in vitro. Using a murine adoptive transfer model, where B cells that are isogenic for the iGL-VRC01 BCR were transferred into wild-type mice, we showed that a priming immunization with iv4/iv9 was capable of engaging and expanding iGL-VRC01 B cells in vivo when the target B cells were present at supraphysiological frequencies^34^.

VRC01 class antibodies undergo extensive somatic mutation to achieve their remarkable breadth and potency. In particular, mutation of the chromosomally encoded CDRL1 to increase flexibility of, or shorten the loop, is often selected to accommodate a highly conserved glycan present at the N276 position on Env^7,8^. Alternatively, mutations outside of the CDRL1 can improve overall affinity and overcome the steric barrier posed by the 276 glycan^35,36^. Somatic hypermutation occurs in germinal centers (GCs) where B cells compete for antigen, and T cells help in a Darwinian process that selects for high-affinity BCRs^37^. It has been proposed that a sequential vaccine regimen utilizing a germline targeting immunogen to first prime unmutated VRC01 precursor B cells, followed by boosting with native-like immunogens, will drive maturation in GCs and consequently increase potency and breadth of the BCRs^1,38^. The success of a prolonged regimen will require effective competition between VRC01 class B cells and off-target B cells in GCs, the establishment of a memory response or long-term germinal centers, and ultimately long-lived plasma cells that secrete protective antibodies. The relative abundance of VRC01 class B cells and their affinity to the immunogen dictate how well these cells compete against off-target B cells in small animal models^39–46^. In addition, circulating serum antibodies can differentially affect B-cell responses to antigen. High-affinity epitope-specific antibodies can block epitope-focused responses, while more diverse, lower-affinity responses can enhance B cell responses^47–59^.

We previously hypothesized that priming with an ai-mAb that is antigenically distinct from Env would selectively target VRC01-class precursor B cells while avoiding the activation of off-target Env-reactive B cells that give rise to non- or narrowly neutralizing antibodies^32^. In this scenario, we proposed that target B cells will expand, such that they can compete more effectively with abundant off-target B cells during subsequent immunizations with more native-like Env immunogens. Here, we tested this hypothesis using the adoptive transfer model we previously leveraged to demonstrate iv4/iv9 priming of iGL-VRC01 B cells^34,40^. Mice were primed with either the bispecific iv4/iv9 ai-mAb^34^ or a germline-targeting HIV-1 Env protein engineered to engage VRC01 class precursors; 426.Mod.Core (426c.NLGS.TM4ΔV1-3 in ref. ^28^). Both groups of mice were subsequently immunized a second time with the germline-targeting Env. A single immunization with either prime immunogen readily expanded VRC01 B cells and promoted their entry into germinal centers. After both groups received a subsequent immunization with Env, a much higher frequency of VRC01 class B cells was observed in mice primed with Env as compared to those primed with the ai-mAb. Analysis of BCR sequences from B cells primed with the non-Env-derived ai-mAb revealed that this immunogen drove somatic mutation away from Env recognition. We further demonstrate that passive delivery of vaccine-elicited, Env-specific non-iGL-VRC01 IgG following an ai-mAb prime could promote expansion and GC entry of iGL-VRC01 class B cells following an Env boost to a comparable level as seen in mice immunized twice with Env. The improvement of on-target GC responses, dependent on the presence of off-target IgG, provides evidence for a positive antibody feedback mechanism. Collectively, our results disfavor the use of antigenically disparate non-Env immunogens in prime boost regimens and inform the design of sequential vaccine regimens designed to elicit broadly neutralizing antibodies against HIV-1.

## Results

### Optimizing an adoptive transfer model to test bispecific iv4/iv9

Using an adoptive transfer model where 500,000 iGL-VRC01 cells expressing the CD45.2 allele were engrafted into Wild-type (WT) mice expressing the CD45.1^+^ allele, we previously demonstrated that immunization with a bispecific germline targeting ai-mAb, iv4/iv9, formulated with sigma adjuvant system (SAS), successfully activated target B cells, initiated antigen-specific GC responses, and generated low serum binding titers to the VRC01 class germline-targeting immunogen eOD-GT8^34^. However, under these conditions, we did not observe an increase in the overall frequency of transferred CD45.2^+^ cells after iv4/iv9 immunization^34^.

**Fig. 1:**
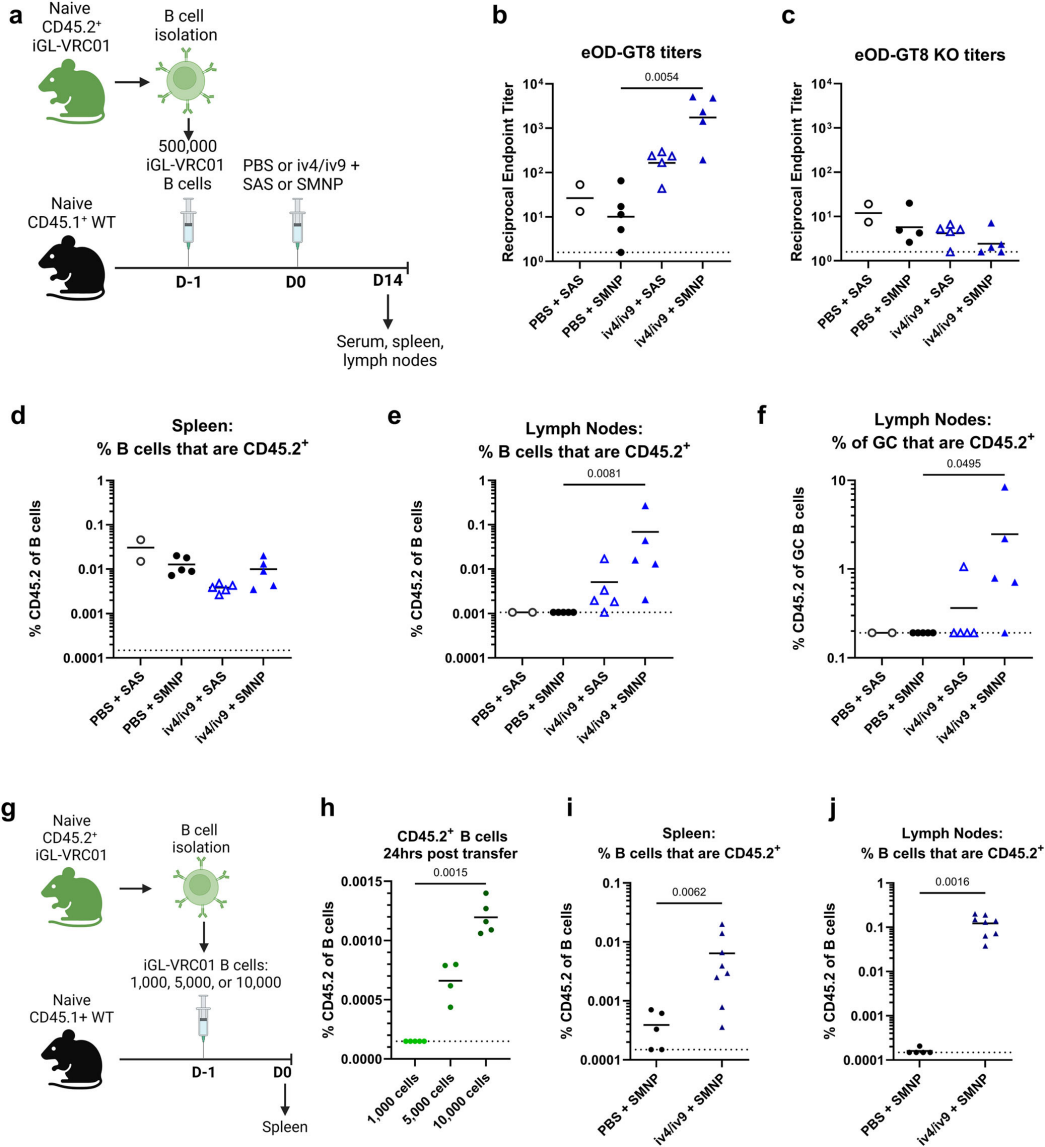
Optimizing immunization with iv4/iv9 in an adoptive transfer model. **a**–**f** CD45.1^+^ wild-type mice (WT) received 500,000 CD45.2^+^ iGL-VRC01 cells, followed by immunization with iv4/iv9 or PBS formulated with either sigma adjuvant system (SAS) or Saponin/Monophosphoryl Lipid A nanoparticle (SMNP) the next day as indicated. Blood, spleens, and lymph nodes were collected 14 days later (**a**). ELISA was used to measure total serum IgG titers against eOD-GT8 (**b**) and eOD-GT8 KO (**c**). Frequency of CD45.2^+^ cells as proportion of total B cells in the spleen (**d**) or lymph nodes (**e**). Frequency of CD45.2^+^ cells as a proportion of germinal center (GC) cells in the lymph nodes (**f**). **g** CD45.1^+^ mice received indicated number of CD45.2^+^ iGL-VRC01 cells. **h** 24 h later, the frequency of CD45.2^+^ cells present in the spleens was analyzed by flow cytometry. *P* values are reported as determined by the Kruskal–Wallis test with Dunn’s multiple comparisons, and the dashed line indicates half the lowest dilution tested. **i** and **j** CD45.1^+^ WT mice received 5000 CD45.2^+^ iGL-VRC01 cells and were immunized 24 h later with iv4/iv9 + SMNP or PBS + SMNP. The frequency of CD45.2^+^ cells was measured in the spleen (**i**) or lymph nodes (**j**) 14 days later. Each data point represents one mouse. *P* values determined by Mann-Whitney tests. **a** and **g** were created using BioRender.

Therefore, we investigated whether formulation of iv4/iv9 with another adjuvant could more efficiently expand target B cells in the same adoptive transfer model. We evaluated SMNP, a nanoparticle adjuvant derived from saponin and monophosphoryl Lipid A, known to elicit larger GC and serum antibody responses than SAS^60^. Wild-type (WT) mice received 500,000 CD45.2^+^ iGL-VRC01 B cells^34,40^ on day -1 (D-1) (Fig. 1a). The next day (D0), they were immunized via intra-muscular (I.M.) injection in the quadriceps with PBS (negative control) or iv4/iv9, formulated with either SAS or SMNP. 14 days later, VRC01-class serum titers were assessed by measuring binding to eOD-GT8 and eOD-GT8 with mutations that disrupt VRC01 class binding (eOD-GT8 KO) by ELISA^30^. Additionally, CD45.2^+^ B cell populations were monitored in the spleen and lymph nodes using flow cytometry. Immunization with iv4/iv9 formulated with SMNP elicited eOD-GT8 serum antibody binding titers that were ~90-fold higher than those elicited with SMNP alone, while there was no statistical difference between iv4/iv9 formulated with SAS and SAS alone (Fig. 1b). All mice had low to absent titers to eOD-GT8 KO, indicating that the eOD-GT8-binding serum antibodies were against the CD4-BS (Fig.1c). Immunization of WT CD45.1 mice, lacking iGL-VRC01 B cells, with iv4/iv9 did not elicit eOD-GT8-binding serum antibodies (Supplementary Fig. 1), confirming that the measured eOD-GT8 titers arose from the transferred iGL-VRC01 cells (Fig. 1c). Neither regimen expanded the frequency CD45.2^+^ B cells in the spleen (Fig. 1d), while formulation with SMNP led to a significant expansion in the lymph nodes (LN, Fig. 1e). CD45.2^+^ B cells were observed among germinal center (GC) B cells in the LN of 4/5 mice immunized with iv4/iv9 + SMNP, while this population was only present in 1/5 mice immunized with iv4/iv9 formulated with SAS (Fig. 1f). Collectively, these data show that formulation of iv4/iv9 with SMNP elicits superior CD45.2^+^ iGL-VRC01-origin B cell and serum antibody responses as compared to SAS.

Transfer of 500,000 CD45.2^+^ iGL-VRC01 B cells results in a frequency of ~1 in 20,000 among B-splenocytes at the time of immunization 24 h later^34^. This is likely supraphysiological since estimates of the frequency of VRC01 precursor B cells in humans range from ~1 in 10,000 to ~1 in 500,000^30,61–63^. We therefore sought to establish transfer conditions under which CD45.2^+^ iGL-VRC01 B cells are present at a more physiological frequency. We transferred 1000, 5000, or 10,000 CD45.2^+^ iGL-VRC01 B cells into CD45.1^+^ WT mice and measured their frequency in the spleen one day later (Fig. 1g). Although we were unable to reliably detect CD45.2^+^ cells when 1000 were transferred, delivery of 5000 and 10,000 cells resulted in measurable frequencies of 0.006%, or ~1 in 200,000 and 0.001% or ~1 in 100,000 B cells, respectively (Fig. 1h).

We next assessed whether iv4/iv9 + SMNP could effectively stimulate CD45.2^+^ iGL-VRC01 B cells when they are present at a more physiological frequency. CD45.1^+^ WT mice received 5000 CD45.2^+^ iGL-VRC01 cells and were immunized with iv4/iv9 + SMNP or adjuvant alone 24 h later. 14 days post immunization, we observed a 16-fold expansion of CD45.2^+^ B cells in the spleen that was statistically significant (Fig. 1i), and a significant expansion of CD45.2^+^ B cells in the LN (Fig. 1j) relative to the adjuvant control, confirming successful targeting of CD45.2^+^ iGL-VRC01 B cells. Given these results, all subsequent adoptive experiments were carried out in mice that received 5000 CD45.2^+^ iGL-VRC01 cells and immunizations were formulated with SMNP as adjuvant.

### Comparing B cell responses to homologous and heterologous prime boost regimens

**Fig. 2:**
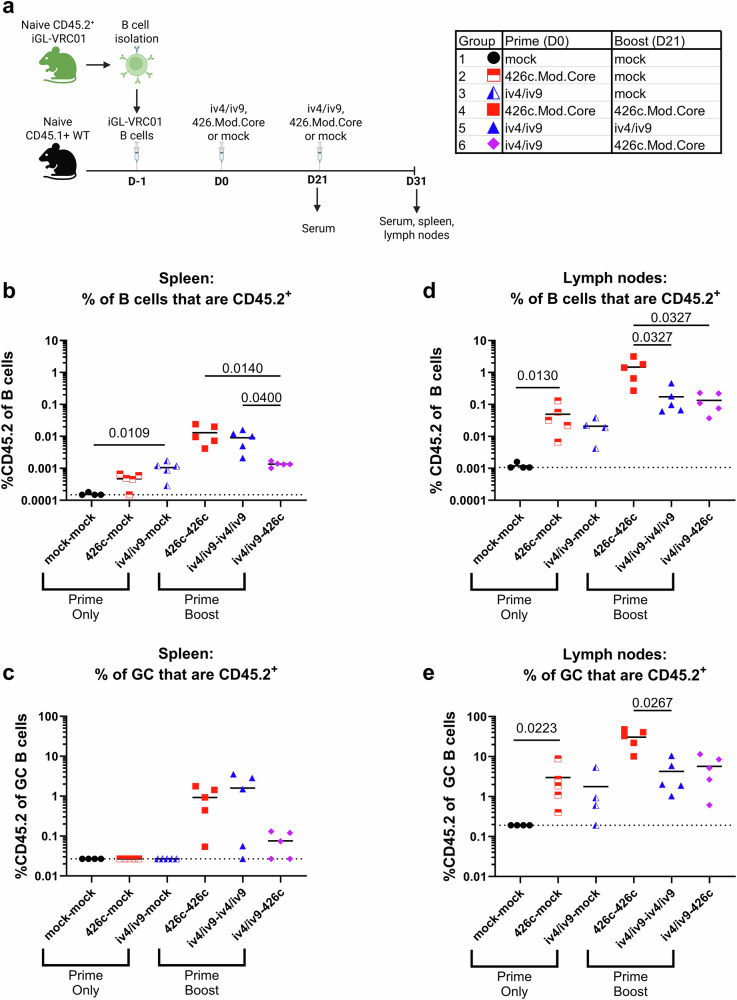
B cell responses to prime—boosting with iv4/iv9 and 426c. **a** Schematic of the experiment. CD45.1^+^ mice received 5000 CD45.2^+^ iGL-VRC01 cells. The next day (D0), they were immunized with either iv4/iv9-2W1S or 426c.Mod.Core-2W1S formulated with SMNP (*n* = 5 per group) or mock immunized (PBS + SMNP, *n* = 4 per group). 21 days later, the mice were bled and immunized a second time with the indicated immunogen. The mice were then euthanized 10 days later (D31). Created with BioRender. **b** Frequency of CD45.2^+^ cells as a proportion of total B cells in the spleen. **c** Frequency of CD45.2^+^ cells as a proportion of germinal center (GC) B cells in the spleen. **d** Frequency of CD45.2^+^ cells as a proportion of total B cells in the lymph nodes. **e** Frequency of CD45.2^+^ cells as a proportion of germinal center (GC) B cells in the lymph nodes. Each data point represents one mouse, bars represent the mean, and dashed lines indicate the limit of detection (LoD) in (**b–e**). *P* values are reported as determined by the Kruskal–Wallis test with Dunn’s multiple comparisons within each group. Statistical tests were performed separately on the Prime Only and Prime Boost groups, indicated by brackets in (**b**–**e**).

Next, we sought to test whether priming with a germline targeting ai-mAb would expand an initial pool of VRC01 class B cells and avoid off-target B cell responses directed at non-VRC01 epitopes on an Env-derived immunogen. We further sought to test whether this strategy would confer a competitive advantage to VRC01-class B cells over the more abundant off-target B cells during subsequent immunizations with a conventional germline targeting Env-derived immunogen. CD45.1^+^ WT mice harboring CD45.2^+^ iGL-VRC01 cells were immunized with either iv4/iv9, 426c.Mod.Core (herein called 426c), or PBS + SMNP (mock). Three weeks later, they received a second immunization with the same immunogen used in the prime (homologous boost), while one group of animals primed with iv4/iv9 were boosted with 426c (heterologous boost, Fig. 2a). Both immunogens were C-terminally tagged with a linear peptide, 2W1S, which is a known CD4+ T cell epitope in the C57BL/6 background^64^. We previously used this peptide fusion strategy to improve B cell responses elicited by an ai-mAb^32^ and reasoned that conjugating this peptide to both iv4/iv9 and 426c would ensure that there was at least one shared helper T cell epitope between the prime and boost immunogens to facilitate the acquisition of help from memory CD4^+^ T cells. Additional groups of animals were primed with iv4/iv9 or 426c at D0 but mock boosted with PBS + SMNP and were analyzed at day 31 as controls.

A single immunization with 426c or iv4/iv9 followed by mock boost led to a slight expansion of CD45.2^+^ B cells in the spleen measured at the time of euthanasia (D31, Fig. 2b). A second immunization with the same antigen used in the prime (homologous boost) further increased the frequency of CD45.2^+^ B cells to comparable levels in the spleen (Fig. 2b). The 426c homologous boost regimen induced splenic CD45.2^+^ GC responses (Fig. 2c), while boosting with 426c did not increase the frequency of CD45.2^+^ B cells that were primed by iv4/iv9 in the spleen (Fig. 2b), however a small population of splenic CD45.2^+^ GC B cells was present in some animals following the iv4/iv9 prime- 426c boost (Fig. 2c).

In the lymph nodes, a single immunization with either iv4/iv9 or 426c followed by a mock boost, led to a slight expansion of CD45.2^+^ B cells (Fig. 2d) and induction of CD45.2^+^ GC responses (Fig. 2e). For both antigens, a homologous boost further expanded CD45.2^+^ cells (Fig. 2d). This expansion was significant and ~10-fold higher in the homologous 426c prime-boost regimen as compared to the homologous iv4/iv9 prime-boost regimen (Fig. 2d). The homologous 426c regimen also elicited the highest frequency of CD45.2^+^GC B cells in the lymph nodes compared to the iv4/iv9 homologous regimen (Fig. 2e). The frequency of CD45.2^+^ B cells among all (Fig. 2d) and GC (Fig. 2e) lymph node B cells in the heterologous iv4/iv9–426c regimen were comparable to the homologous iv4/iv9 regimen.

### ai-mAb immunization induces somatic mutations that negatively impact 426c recognition by iGL-VRC01 B cells

**Fig. 3:**
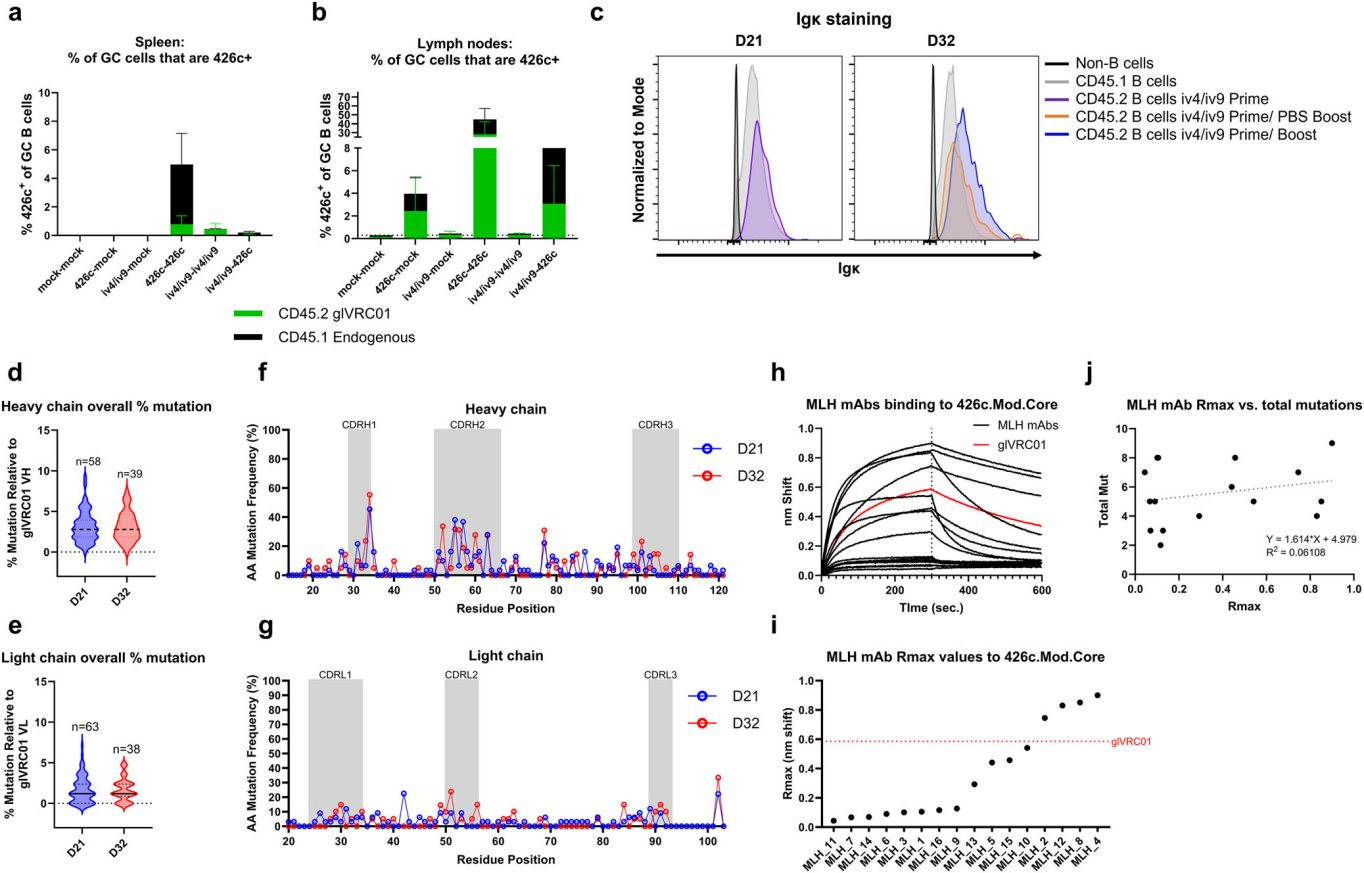
BCR binding to 426c.Mod.Core following ai-mAb immunization and sequence analysis of CD45.2 LN GC + B cells following ai-mAb immunization. **a** and **b** Proportion of GC B cells from mice in Fig. 2 that stain 426c.Mod.Core^+^ in spleen (**a**) and lymph nodes (**b**); the height of the bars indicates the mean total frequency of 426c.Mod.Core^+^ GC cells, and the colors indicate whether they are of CD45.1^+^ (black) or CD45.2^+^ (green) origin. Bars represent the mean of *n* = 5 mice per group, and the error bars show the standard deviation. **c** Overlaid histograms showing IgK staining by flow cytometry of indicated cell populations at the indicated timepoints. Data normalized to mode. **d–g** Sequence and mutation analysis of single-cell sorted CD45.2^+^ B cells from the lymph nodes of iv4/iv9 prime, iv4/iv9 boost animals at D21 and D32. **d** and **e** Violin plots of overall percent mutation in the heavy chain (IgH, **d**) or light chains (IgK, **e**) compared to iGL-VRC01 with the number of chains analyzed above each plot. **h–j** Binding of 16 recombinant mAbs corresponding to BCRs from sorted cells was assessed for binding to a 500 nM solution of 426c.Mod.Core by BLI. **h** Representative BLI traces of sorted mAbs (black) compared to iGL-VRC01 IgG (red). **i** *R*_max_ values of sorted mAbs to 426 c.Mod.Core from (**h**). *R*_max_ of iGL-VRC01 to 426c.Mod.Core shown by the red dotted line. **j** Linear regression analysis of *R*_max_ values from (**i**) vs. number of mutations relative to iGL-VRC01.

To quantify on-target CD45.2^+^ B cells of iGL-VRC01 origin and off-target CD45.1^+^ 426c-specific GC B cell responses of WT origin, we stained GC B cells collected from the spleen and lymph nodes from the mice in Fig. 2 at D31 with 426c-tetramers, using a dual-labeling strategy. In the spleen, we observed the highest frequency, ~6%, of 426c ^+^ GC B cells in the group that received two immunizations with 426c (Fig. 3a). A minority of these, ~0.8%, were CD45.2^+^ (Fig. 3a). In mice immunized with a homologous iv4/iv9 prime-boost regimen, ~0.3% of the splenic GC B cells were 426c^+^ and nearly all were CD45.2^+^. The heterologous iv4/iv9-426c regimen elicited a very low frequency of 426c ^+^ B cells, most of which were off-target CD45.1 cells (Fig. 3a). The low frequency of CD45.2^+^ cells among 426c^+^ B cells in the iv4/iv9-426c regimen is consistent with smaller expansion of total CD45.2^+^ GC B cells in this group as compared to the homologous prime-boost groups (Fig. 2c).

In the lymph nodes, the 426c-426c regimen elicited the highest frequency of total antigen-specific GC B cells, ~45%, most of which (~30%) were CD45.2^+^ (Fig. 3b). In comparison, the heterologous iv4/iv9 prime-426c boost yielded a lower frequency of 426c ^+^ GC B cells in the lymph nodes (~7%), with the majority being off-target (Fig. 3b). Although the iv4/iv9-iv4/iv9 and iv4/iv9-426c regimens led to a comparable expansion of CD45.2^+^ B cells in the LN GCs at D31 (Fig. 2e), 426c^+^/CD45.2^+^ LN GC B cells were not detected in mice immunized twice with iv4/iv9 (Fig. 3b) suggesting that immunization with iv4/iv9 negatively affects subsequent CD45.2^+^ B cell binding to 426c. In support of this notion, we observed that only ~30% of splenic CD45.2^+^ B cells in mice immunized twice with iv4/iv9, bound 426c, while ~70–80% bound 426c in all other groups (Supplementary Fig. 2a). Similarly, ~70–80% of lymph node CD45.2^+^ B cells bound 426c in animals immunized once or twice with 426c while only ~20% bound when the animals were immunized once or twice with iv4/iv9 (Supplementary Fig. 2b).

To better understand the mechanism underlying the reduced binding of CD45.2^+^ GC B cells to 426c following iv4/iv9 immunization, we repeated the iv4/iv9 prime only, and iv4/iv9 prime-boost immunizations (i.e., Fig. 2a, Groups 3 and 5) and focused our analyses on LN B cells. Igκ staining was observed on non-iGL VRC01 (CD45.1^+^) and CD45.2^+^ iGL-VRC01 origin cells at the time of the second immunization (D21) or 11 days after a second immunization (D32), indicating that the reduced 426c binding observed in GC LNs is not attributed to loss of BCR expression on the cell surface (Fig. 3c).

Lymph node cells from iv4/iv9 immunized mice were pooled, and CD45.2^+^ GC B cells were subjected to antigen-unbiased single-cell sorting (Supplementary Fig. 3), and their variable heavy (VH) and variable light (VL) BCR transcripts were recovered by RT-PCR and sequenced. Among all recovered transcripts, we observed higher frequencies of heavy chain mutations as compared to light chain mutations with no statistical differences in these frequencies between the time of the boost (D21) and 11 days later (D32, Fig. 3d and e). Most of the mutations were found in the CDR1 and CDR2 regions of the heavy and light chains (Fig. 3f and g, Supplementary Fig. 4, and Supplementary Tables 2-4). 22/62 or 35% of paired sequences had at least one mutation found in VRC01 class antibodies. We randomly selected 16 representative heavy and light chain pairs (roughly 25% of the total pairs recovered), four from each quartile based on the HC mutation rate (Fig. 3d and e), isolated at either D21 or D32 (see Supplementary Fig. 4 for more details). This included some pairs that had mutations found in mature VRC01 (Supplementary Figs. 4 and 5, and Supplementary Tables 2–4). These pairs were produced as recombinant mAbs and evaluated for binding to 500 nM of 426c by biolayer interferometry (BLI). This analysis revealed that some mAbs clearly had reduced, or absent binding to 426c as compared to iGL-VRC01 with discernible differences in the association and dissociation rates (Fig. 3h). Using the maximum response (*R*_max_) measured at the end of the dissociation phase as a proxy for binding affinity we found that only 4/16 (25%) had stronger binding than iGL-VRC01 (Fig. 3i). 8 (50%) had negligible binding to 426c, while 4 (25%) retained some binding Fig. 3i). There was no obvious correlation between *R*_max_ and the frequency of total (Fig. 3j), heavy (Supplementary Fig. 6a), or light (Supplementary Fig. 6b) chain mutations. 4 of the mAbs had mutations in VH1-2*02 encoded contact residues, W50, N58, R71, and W100B found in potent VRC01 like antibodies as defined in ref. ^65^ (Supplementary Fig. 4a), all of which showed near-complete abrogation of 426c binding (Supplementary Fig. 6c). Among the recombinant mAbs we produced, 4 acquired heavy chain mutations found in mutated VRC01: MLH_2 G30D, MLH_4 Y91F, MLH_6 Q105R and MLH_11 I51L (Supplementary Fig. 4a). Among these, only MLH_2 and MLH_4 showed enhanced binding to 426c.Mod.Core, while the binding of the other two was reduced (Fig.3i). Among the mAbs that bound 426c.Mod.Core only one, MLH_2 acquired a key VRC01 class mutation (G31D), but it lost a germline-encoded germline contact residue (R66W), Supplementary Fig. 5 and Supplementary Tables 2 and 3). None of the mAbs that lost affinity acquired key VRC01-class mutations (Supplementary Figs. 4 and 5, and Supplementary Tables 2 and 3). Interestingly, mAbs that lost affinity to 426c do not appear to gain affinity towards iv4 or iv9, but most maintain binding as determined by BLI (Supplementary Fig. 6d–h).

Collectively, these analyses reveal quantitative and qualitative differences in the frequencies of CD45.1 and CD45.2 B cell responses measured in the spleen and lymph nodes. They also indicate that in this model, the homologous 426c prime-boost regimen, despite eliciting off-target B cell responses, elicited a higher frequency of on-target B cell responses (total and GC) as compared to a homologous iv4/iv9, or heterologous iv4/iv9-426c boost. These data also indicate that immunization with the ai-mAb induces BCR mutations that negatively impact binding to 426c.

### Comparing plasma responses to homologous and heterologous prime-boost regimens

**Fig. 4:**
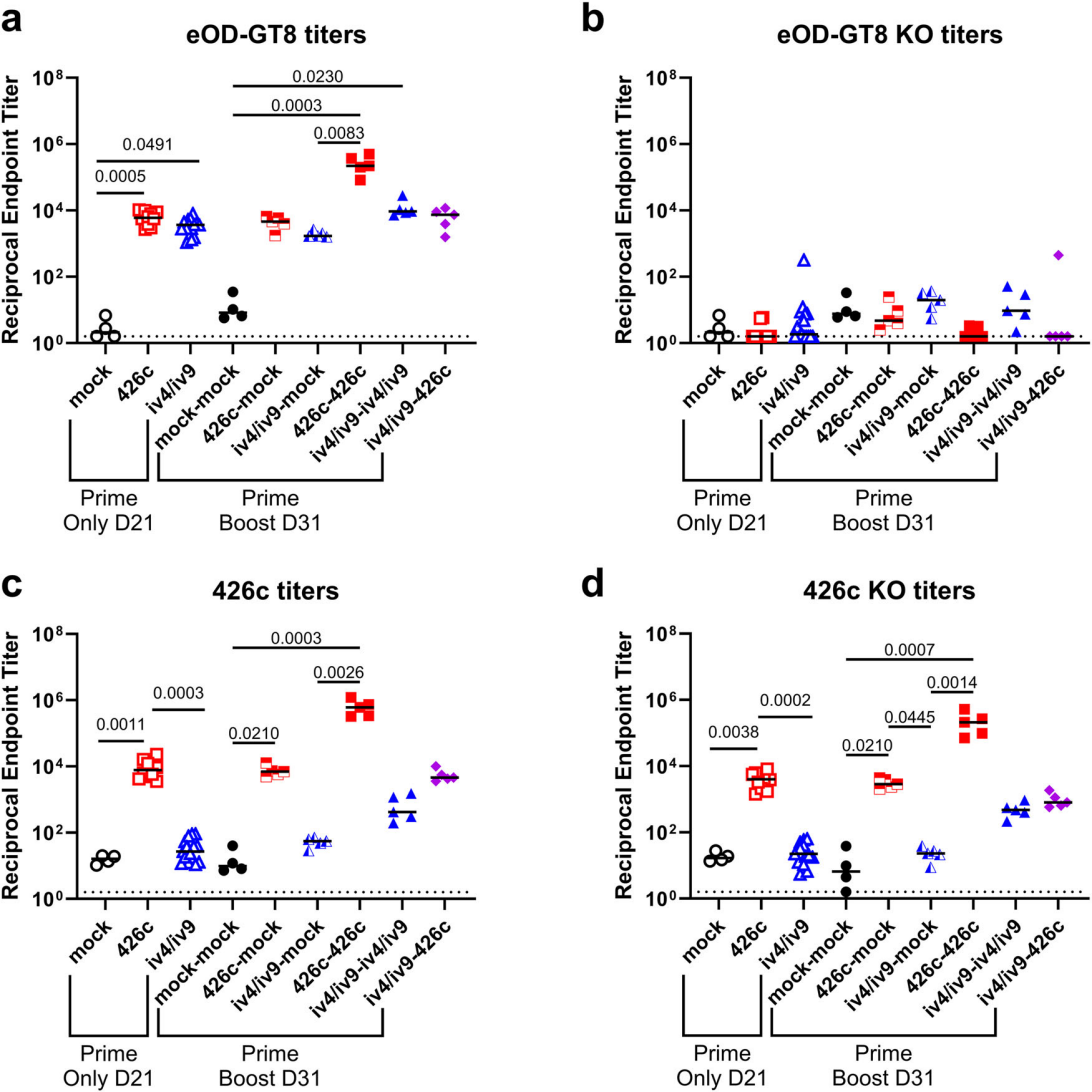
Serum responses to 426c and eOD-GT8 antigens in immunized mice. **a–d** Serum was collected at D21 and D31 from the experiment shown in Fig. 2 and analyzed by ELISA. Endpoint binding titers were measured to eOD-GT8 (**a**), eOD-GT8-KO (**b**), 426c.Mod.Core (**c**), and 426c.Mod.Core-KO (**d**). Dashed line indicates half of the lowest dilution tested. Each data point represents one mouse. *n* = 5 mice per group, except the PBS only control group, where *n* = 4 mice. Mean is plotted. *P* values are reported as determined by the Kruskal–Wallis test with Dunn’s multiple comparisons. Statistical tests were performed separately on the Prime Only and Prime Boost groups, indicated by brackets.

To assess both circulating VRC01-epitope-specific (on-target) and non-epitope-specific (off-target) antibody responses from immunized animals in Fig. 2, serum binding titers to eOD-GT8 and eOD-GT8-KO, as well as 426c and 426c-KO were measured by ELISA (Fig. 4). A single immunization with either iv4/iv9 or 426c elicited epitope-specific antibodies to eOD-GT8 (D21, Fig. 4a). In contrast, only mice immunized with 426c showed strong binding titers to 426c (Fig. 4c) after a single immunization. We note that the affinity of iGL-VRC01 for eOD-GT8 is in the pM range^30^ while 426c is 0.3µM^28^, making eOD-GT8 a more sensitive reagent for detecting serum responses in this assay. The same mice had slightly lower (~2-fold) titers to 426c-KO (Fig. 4d and Supplementary Fig. 7). Control (CD45.1) mice that were immunized with 426c without receiving CD45.2^+^ iGL-VRC01 B cells had lower plasma antibody binding titers to 426c (~2.6-fold) and 426c-KO (~2.2-fold) relative to those harboring iGL-VRC01 (Supplementary Fig. 7). The titers were ~1.8-fold lower to 426c-KO relative to 426c in the no transfer control group (Supplementary Fig. 7). Collectively these data indicate that a substantial fraction of the circulating antibodies in 426c immunized mice are directed at off-target non-CD4-BS epitopes irrespective of the presence of CD45.2^+^ iGL-VRC01 B cells.

Titers to eOD-GT8 significantly increased in groups primed with either iv4/iv9 or 426c followed by a homologous boost (Fig. 4a). However, titers to 426c only significantly increased in animals that received homologous 426c prime-boost, while those that received iv4/iv9 homologous prime-boost increased but did not reach statistical significance (Fig. 4c). In contrast, mice which received an immunogen at D0 but were mock-boosted with PBS + SMNP at D21 had unchanged titers to eOD-GT8 and 426c at D31 confirming that the measured changes in serum titers at D31 were due to the second immunization with either iv4/iv9 or 426c (Fig. 4a and c). The homologous 426c prime-boost regimen elicited the highest binding titers to eOD-GT8, and to both 426c and 426c-KO (Fig. 4a, c, and d), consistent with the expansion of both on-target and off-target B cell populations among GC B cells in the lymph nodes (Figs. 2e and 3b).

Animals that received a heterologous iv4/iv9 prime-426c boost regimen had considerably higher 426c-specific titers compared to animals that were only primed with iv4/iv9 (Fig. 4c). However, the titers to 426c-KO also increased after the boost in the iv4/iv9prime-426c-boost group (Fig. 4d), while the eOD-GT8 titers remained unchanged (Fig. 4a), suggesting that most of the serum responses elicited by the second 426c immunization were de novo responses directed at off-target epitopes.

### Circulating on-target antibodies modestly affect the priming of iGL-VRC01 B cells

Similar adoptive transfer systems have demonstrated that circulating antibodies can inhibit or enhance GC populations of naive B cells depending on BCR affinity, epitope specificity and antibody titer^48,56^. We observed that priming with iv4/iv9 and 426c both elicited high titers of on-target iGL-VRC01-origin antibodies (Fig. 4a), while the latter also elicited high titers of off-target non-VRC01 antibodies, which includes those that bind outside and within the CD4-BS (Fig. 4d and Supplementary Fig. 7). We therefore sought to investigate the influence of these circulating on- and off-target antibodies on the B cell recall responses elicited upon boosting in this model.

**Fig. 5:**
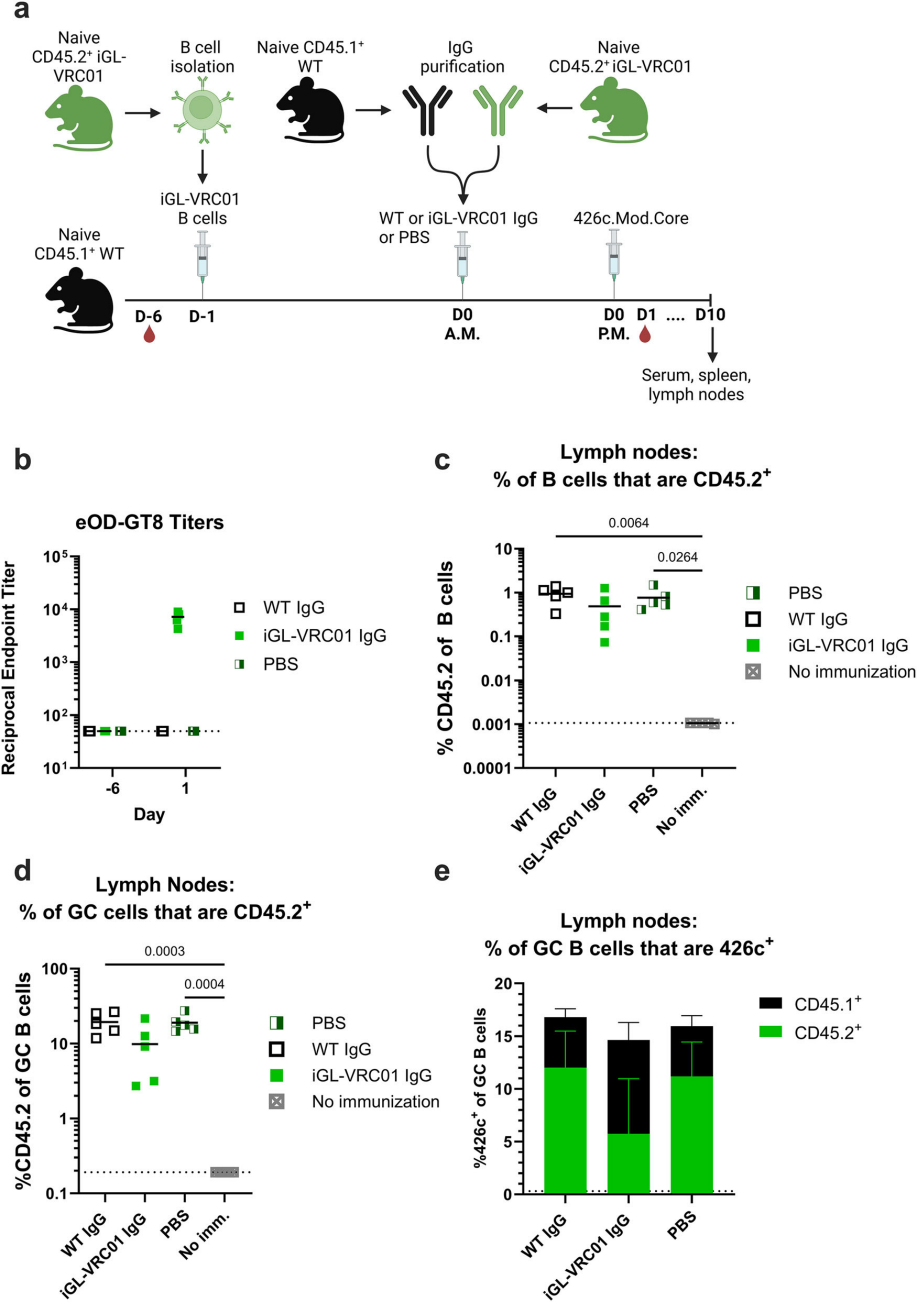
Effect of on-target antibodies on B cell responses to a 426 c.Mod.Core prime. **a** Schematic of the experiment. CD45.1^+^ WT mice (*n* = 5 per group) received 5000 CD45.2^+^ iGL-VRC01 cells (D-1). The next morning (D0 A.M.), they received IgG purified from naïve CD45.1^+^ WT mice, iGL-VRC01 mice or PBS, followed by an immunization with 426c.Mod.Core in the afternoon (D0 P.M.). Created with BioRender. **b** Endpoint binding titers to eOD-GT8 were measured by ELISA in serum collected from the indicated groups on D-6 and D1. Dashed line indicates half the lowest dilution tested. **c** Frequency of total B cells that are CD45.2^+^ in the lymph nodes at day 10. **d** Frequency of germinal center (GC) B cells that are CD45.2^+^ in the lymph nodes at day 10. Each data point represents one mouse (*n* = 5), and the mean is indicated by a bar. *P* values are reported as determined by the Kruskal–Wallis test with Dunn’s multiple comparisons. Dashed lines indicate the limit of detection in (**b**–**d**). **e** Mean percentage of 426c.Mod.Core^+^ GC B cells in the lymph node at day 10. The height of the bar indicates the total frequency of 426c.Mod.Core^+^ cells, and the colors indicate whether they are CD45.1^+^ (black) or CD45.2^+^ (green). Bars represent the mean of *n* = 5 mice per group, and the error bars represent the standard deviation in (**e**).

We first investigated whether elevated titers of circulating iGL-VRC01 antibodies impacted the ability of the CD45.2^+^ iGL-VRC01 B cells to respond to immunization with 426c. A pre-bleed was collected on recipient WT CD45.1^+^ mice; and then 6 days later, they received CD45.2^+^ iGL-VRC01 B cells on day -1 (D-1). The next morning (D0, A.M.), they received PBS, or 500 µg of IgG purified from naive iGL-VRC01 mice or naïve wildtype mice. ~8 hours later (D0, P.M.), they were immunized with 426c. The timing of the IgG immunization and transfer was done to minimize the effects of antibody decay from serum and is based on a similar adoptive transfer model^48^. Serum was then collected on D1, and the mice were euthanized nine days later (Fig. 5a).

Sera from mice that received iGL-VRC01 IgG had no eOD-GT8 binding titers at baseline (D-6); while at one day post transfer, they were comparable to those observed at day 21 in mice primed with either iv4/iv9 or 426c alone (compare Figs. 5b and 4a), indicating that the IgG transfer was successful. 10 days after 426c-immunization, we observed a comparable frequency of CD45.2^+^ B cells in the lymph nodes of animals that received either PBS, or control WT IgG (Fig. 5c). Mice which received iGL-VRC01 IgG had marginally lower frequencies of CD45.2^+^ cells in the lymph nodes; both as a proportion of total B cells and of GCs as compared to the control groups, however the differences were not statistically significant (Fig. 5c and d).

The frequency of 426c-specific GC B cells was comparable in all 3 groups at D10 (Fig. 5e). ~66% of these were CD45.2^+^ B cells in the PBS and control IgG transfer groups, while only ~33% were CD45.2^+^ B cells in mice that received iGL- VRC01 IgG prior to immunization (Fig. 5e). We did not observe any difference in the frequency of iGL-VRC01 B cells in the spleens of immunized mice relative to unimmunized mice (Supplementary Fig. S8c–e). Collectively, these results indicate that under these experimental conditions, pre-existing iGL-VRC01 serum antibodies had a modest inhibitory effect on CD45.2^+^ iGL-VRC01 responses in the lymph nodes of immunized animals.

### Circulating on-target antibodies do not affect the ability to boost iGL-VRC01 B cells

**Fig. 6:**
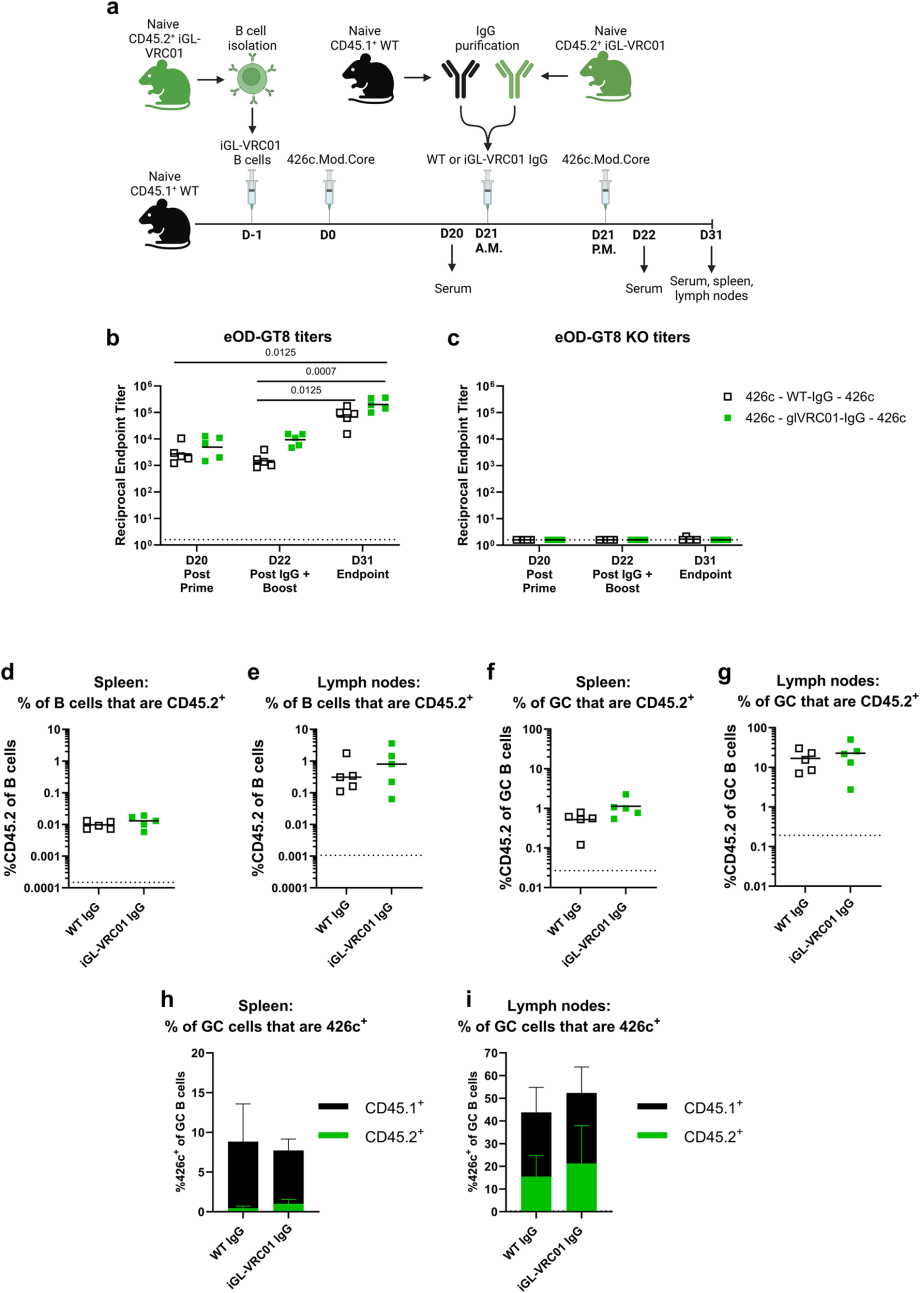
Effect of on-target antibody transfer on response to boost. **a** Schematic of the experiment. CD45.1^+^ WT mice received 5,000 iGL-VRC01 CD45.2^+^ cells and were immunized the next day (D0) with 426c.Mod.Core. Twenty-one days later (D21), they received IgG from naïve CD45.1^+^ WT or from iGL-VRC01 mice in the morning (A.M.), followed by an immunization with 426c.Mod.Core in the afternoon (P.M.). Created with BioRender. **b** and **c** Endpoint binding titers to eOD-GT8 (**b**) or eOD-GT8 KO (**c**) were measured by ELISA in serum collected on D20, D22, and D31. Dashed line represents half of the lowest dilution tested. **d** and **e** Frequency of total CD45.2^+^ B cells in the spleen (**d**) and lymph nodes (**e**) at D31. **f** and **g** Frequency of CD45.2^+^ B cells among GC B cells in the spleen (**f**) and lymph nodes (**g**) at D31. Each data point represents one mouse (*n* = 5). *P* values are reported as determined by the Kruskal–Wallis test with Dunn’s multiple comparisons, and dashed lines indicate limit of detection (**d–g**). **h** and **i** Percentage of 426c.Mod.Core^+^ B cells among GC B cells in the spleen (**h**) and lymph nodes (**i**) at day 31. Bars represent the mean of *n* = 5 mice per group, and the error bars represent the standard deviation. The height of the bar indicates the total frequency of 426c.Mod.Core^+^ cells, and the colors indicate whether they are CD45.1^+^ (black) or CD45.2^+^ (green).

We next asked whether supraphysiological titers of on-target antibodies affect iGL-VRC01 B cell responses to a homologous boost (Fig. 6). Here, CD45.2^+^ iGL-VRC01 cells were transferred into CD45.1^+^ WT mice on D-1. The next day (D0), they were immunized with 426c. Three weeks later (D21), the mice received purified IgG from either naïve WT CD45.1^+^ or CD45.2^+^ iGL-VRC01 mice and were then immunized with 426c a second time (Fig. 6a). Mice which received IgG from iGL-VRC01 donors had higher titers to eOD-GT8 at D22 than those which received WT-IgG (~6 fold higher), while none of the plasma bound to eOD-GT8 KO consistent with successful antibody transfer (Fig. 6b and c).

Following the second immunization with 426c, we observed an increase in eOD-GT8 titers from D22 to D31, in both the control WT IgG and iGL-VRC01 IgG transfer groups (Fig. 6b), indicating that transfer of on-target IgG did not inhibit the serum antibody response to a homologous boost. When we assessed the prevalence of CD45.2^+^ B cells, no differences were observed in the frequencies of total or GC CD45.2^+^ B cells in the spleen or lymph nodes (Fig. 6d–g and Supplementary Fig. S9). Addition of either control WT or iGL-VRC01 IgG resulted in comparable frequencies of total 426c-specific GC B cells in both the spleen and lymph nodes (Fig. 6h and i). Transfer of iGL-VRC01 IgG resulted in a slightly higher proportion of CD45.2^+^ 426c-specific B cells in the spleen and lymph nodes (Fig. 6h and i). Collectively, these data demonstrate that in this experimental setting, administration of supraphysiological levels of iGL-VRC01 IgG following a 426c prime does not negatively affect CD45.2^+^ iGL-VRC01 B cell responses to a homologous boost.

### Circulating off-target antibodies promote on-target iGL-VRC01 B cell responses

**Fig. 7:**
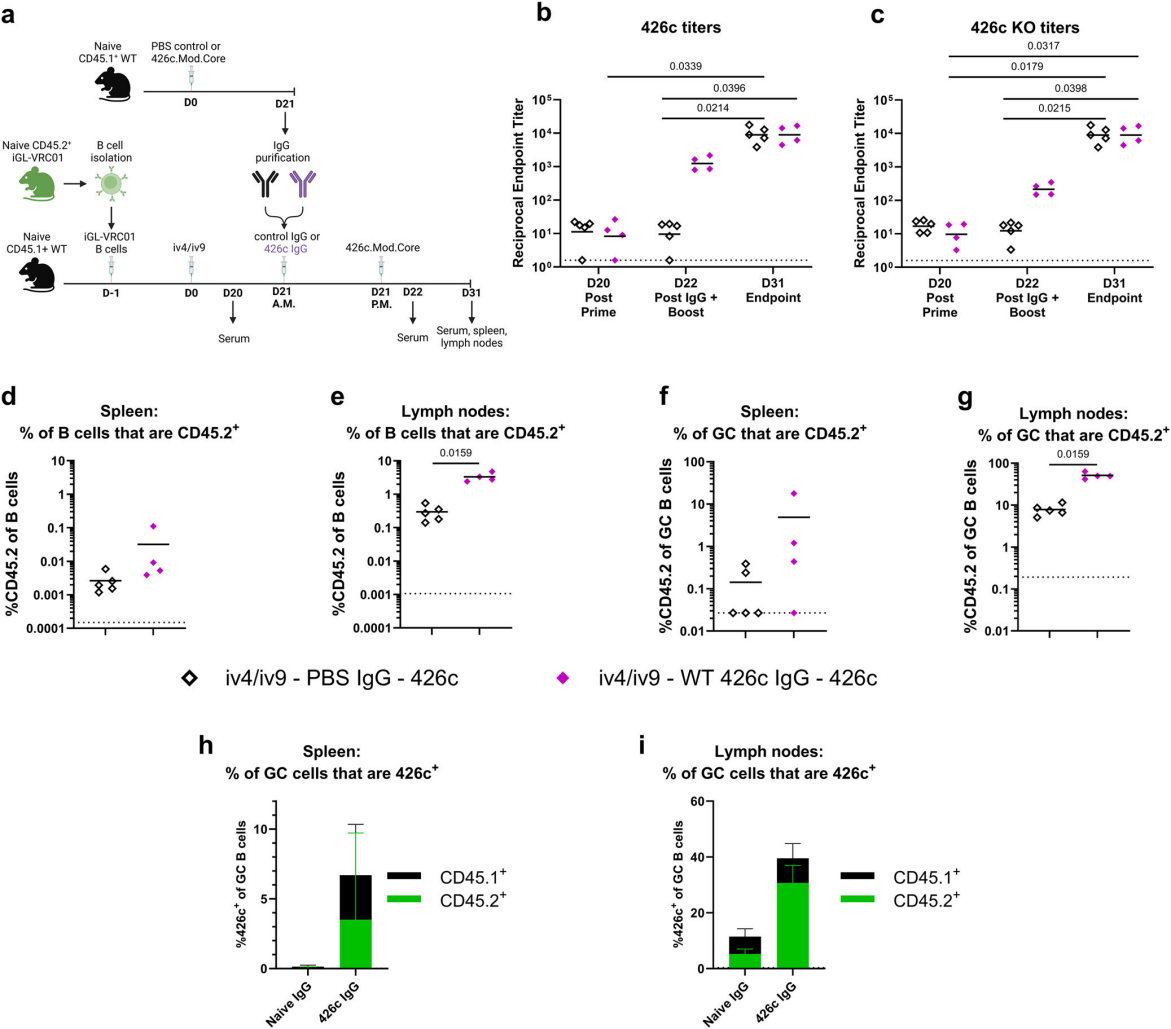
Effect of off-target antibody transfer on response to boost. **a** Schematic of the experiment. CD45.1^+^ wild-type mice received 5000 CD45.2^+^ iGL-VRC01 B cells and were immunized the next day (D0) with iv4/iv9. Twenty-one days later (D21), they received IgG from CD45.1^+^ wild-type mice immunized with PBS + SMNP (control, black *n* = 5) or with 426c.Mod.Core (purple *n* = 4) in the morning (A.M.), followed by an immunization with 426c.Mod.Core in the afternoon (P.M.). Created with BioRender. **b** and **c** Endpoint binding titers to 426c.Mod.Core (**b**) and 426c.Mod.Core KO (**c**) was measured by ELISA in serum collected on D20, D22, and D31. *P* values are reported as determined by the Kruskal–Wallis test with Dunn’s multiple comparisons, and the dashed line indicates half the lowest dilution tested. **d** and **e** Frequency of total CD45.2^+^ B cells in the spleen (**d**) and lymph nodes (**e**) at D31. **f** and **g** Frequency of CD45.2^+^ B cells among germinal center B cells in the spleen (**f**) and lymph nodes (**g**) at D31. Each data point represents one mouse, and bars represent the means. *P* values are reported as determined by the Mann–Whitney test, and the dashed line indicates the limit of detection (**d**–**g**)**. h** and **i** Percentage of 426c.Mod.Core^+^ B cells among GC B cells in the spleen (**h**) and lymph nodes (**i**) at day 31. Bars represent the mean of *n* = 5 mice per group in the naïve IgG group and *n* = 4 in the 426c IgG group, and the error bars represent the standard deviation. The height of the bar indicates the total frequency of 426c.Mod.Core^+^ cells, and the colors indicate whether they are CD45.1^+^ (black) or CD45.2^+^ (green).

To further evaluate the effect of pre-existing antibodies on the iGL-VRC01 B cell response to immunization, we investigated the influence of off-target serum antibodies to 426c on iGL-VRC01 B cell recall responses following an iv4/iv9 prime. To generate off-target antibodies, CD45.1^+^ WT mice lacking CD45.2^+^ iGL-VRC01 B cells were immunized with 426c. Three weeks later, the serum was collected. We verified that both CD4-BS and non-CD4-BS antibodies were present in the serum (Supplementary Fig. 7), and the IgG was subsequently purified. IgG purified from CD45.1^+^ WT mice mock immunized with PBS + SMNP was collected as a control. Experimental groups of CD45.1^+^ WT mice harboring CD45.2^+^ iGL-VRC01 B cells were immunized with iv4/iv9. Three weeks later (D21) in the morning, they received either control IgG from naïve WT mice or off-target 426c-elicited, followed by an immunization with 426c in the afternoon. Serum was collected from the recipients the day before (D20) and the day after (D22) the second immunization (Fig. 7a). Prior to IgG transfer, the 426c and 426c-KO titers were equivalent (Fig. 7b and c). At D22, mice that received 426c -elicited IgG (solid purple diamonds) had higher titers to both 426c and 426c-KO than those that received control IgG (open black diamonds), consistent with a successful transfer of antibody (Fig. 7b and c). At this timepoint, the titers were approximately 10-fold higher to 426c than they were to 426c-KO, indicating that both CD4-BS and non-CD4-BS antibodies were transferred. At the endpoint (D31), the serum titers to 426c and 426c -KO were boosted to equivalent titers in both groups, indicating that the second 426c immunization elicited 426c-specific serum responses that augmented those from the prime and the IgG transfer. The addition of off-target IgG, between the prime and the boost, resulted in a slight increase in the frequency of total and GC CD45.2^+^ B cells in the spleen that was not significant (Fig. 7d and f, and Supplementary Fig. 10). Meanwhile, the addition of off-target IgG resulted in a significantly higher frequency of total and GC CD45.2^+^ B cells in the lymph nodes (Fig. 7e and g and Supplementary Fig. 10). There, almost half of the GC B cells were CD45.2^+^, while only ~5% were CD45.2^+^ in the mice that received control IgG (Fig. 7g).

The addition of off-target IgG, as compared to control IgG also led to a higher total frequency of 426c^+^ GC B cells in the spleen and lymph nodes (Fig. 7h, i). These were dominated by CD45.1^+^ WT B cells in the spleen (Fig. 7h), and by CD45.2^+^ B cells in the lymph nodes (Fig. 7i). The relative proportion of off-target and of CD45.2^+^ cells elicited by the heterologous iv4/iv9-426c regimen in the presence of transferred exogenous off-target IgG mirrored those of the homologous 426c prime-boost regimen (Compare Figs. 3a, b, 7h, i and Supplementary Fig. 11). Collectively, these data indicate that circulating polyclonal off-target antibodies to 426c, including those that bind to the CD4-BS, promote the expansion of on-target iGL-VRC01 class B cells and their recruitment to GCs, particularly in the lymph nodes.

## Discussion

VRC01 class antibodies require high levels of somatic mutation to achieve potent neutralizing breadth, and thus eliciting antibodies of this class through vaccination will almost certainly necessitate a sequential immunization regimen. This will require a prime with a germline targeting immunogen to initiate a VRC01 class B cell response, followed by subsequent immunizations that guide the development of somatic mutations, and in turn, neutralizing breadth and potency. We previously hypothesized that a germline targeting ai-mAb could selectively expand VRC01 class B cells and enable a more efficient boost with 426c by ameliorating anamnestic off-target, non-VRC01 class B cell responses during boost immunizations. Here, we used a murine adoptive transfer model to compare the expansion of on-target VRC01 class and off-target B cell and serum responses using two different germline targeting immunogens: either homologous prime/boost regimens with the ai-mAb or a germline targeting Env-derived immunogen, or a heterologous immunization regimen comprised of an anti-idiotype prime followed by a boost with the germline-targeting Env-derived immunogen. Our findings in this experimental system indicate that an ai-mAb is not an ideal immunogen to prime target B cells in a prolonged prime-boost regimen intended to drive affinity maturation of VRC01 class antibodies for two reasons: (i) the ai-mAb can induce off-target somatic mutation and (ii) it does not elicit off-target 426c-specific serum antibodies that contribute to positive feedback resulting in strong germinal center responses.

Both the ai-mAb and the germline-targeting 426c could reliably engage and induce expansion of iGL-VRC01-B cell responses when precursor B cells were present at a physiological frequency of ~1 in 200,000 B cells, indicating that both could plausibly prime VRC01 class B cell responses in humans. In agreement with our previous study, the overall 426c-specific B cell responses elicited by the ai-mAb prime were entirely of iGL-VRC01 B cell origin, demonstrating selective engagement of on-target VRC01 class B cells and limited off-target 426c-specific B cells^32^. However, through single-cell sorting analyses, we found that most LN iGL-VRC01 GC B cells acquired mutations that reduced or eliminated reactivity with 426c, and only a subset of GC B cells retained the ability to bind 426c. Despite this, at least some B cells in animals primed with iv4/iv9 retained 426c reactivity and were boosted by a subsequent Env immunization. We did not conduct a similar single-cell BCR sequence analysis from mice primed or primed and boosted with 426c. Given the stochastic nature of somatic hypermutation, it is likely that some GC B cells also acquired loss-of-function mutations. However, it is likely that these represent a minority population, given that the majority of iGL-VRC01-origin GC B cells retained 426c binding activity following immunization with 426c.

We found that iGL-VRC01-origin B cell responses primed by iv4/iv9 were enhanced by the presence of circulating polyclonal, off-target 426c-specific antibodies. Thus, even though the ai-mAb prime drove affinity maturation in iGL-VRC01-origin GC B cells away from 426c recognition, the presence of off-target 426c-specific mAbs at the time of a boost immunization with 426c promoted VRC01 class B cell responses to levels comparable to those observed in a 426c-prime, 426c boost regimen, highlighting an example of a powerful positive feedback mechanism. These results are in line with observations that low-affinity, off-target antibody responses can enhance B cell responses^48,56^. In this case, the presence of the pre-existing antibodies is likely leading to antibody–antigen complex formation, as well as complement fixation that facilitates antigen capture and presentation to B cells in lymph node follicles^66–68^. Although our data demonstrate that circulating off-target serum antibodies contribute to the expansion of on-target VRC01 class B cells, additional immune parameters, including T cell help and the establishment of memory B cells, which were not examined here, likely contributed to the overall on- and off-target B cell responses in the prime/boost regimens^54^.

Transfer of cognate iGL-VRC01 antibodies to naïve mice had a marginal effect on iGL-VRC01 B cell responses to a 426c prime. Similarly, introducing supraphysiological levels of iGL-VRC01 antibodies after a 426c prime had no effect on a homologous Env boost, indicating that circulating on-target antibodies from iGL-VRC01 B cells did not exceed a threshold titer to completely inhibit the iGL-VRC01 B cell response to a second immunization, irrespective of the prime^48,56^.

The 426c prime only and the 426c prime-426c boost regimen elicited substantial off-target responses as read out by serum ELISA and 426c-specific GC B cell binding. Nevertheless, iGL-VRC01 class B cells were able to compete in germinal centers demonstrating that 426c can effectively drive VRC01 class responses in the context of a diverse B cell repertoire.

We note that we did not analyze whether antibodies were elicited against the anti-idiotypic mAb. If these were elicited at high titers, they may have negatively affected the ability of the ai-mAb to engage target B cells. The observation that iGL-VRC01 B cells primed with iv4/iv9 were boosted by the same immunogen suggests that any such antibodies were not playing a substantial inhibitory role.

In a similar adoptive transfer system, Tas et al. did not observe GC recruitment of transferred CLK19 VRC01 class B cells 10 days following an eOD-GT8 boost delivered 42 days after an eOD-GT8 prime^48^. In contrast, we observed VRC01 class GC B cells after a prime and an increase in frequency of these cells after a boost on day 21. Given that measurable iGL-VRC01 GC responses were occurring at the time the boost was delivered, it is possible that refueling of the GC responses occurred with the shorter boosting interval, which may explain the differential outcomes between the two studies.

In the experimental system used here, it is not possible to distinguish whether the B cell responses (GC or other) we observe after a second immunization are arising from a recall of memory B cells, an expansion or refueling of cells already in GCs, or recruitment of circulating naïve cells that did not enter the GC on the first exposure to antigen. Moreover, we do not know the frequency of GC B cells exiting and establishing a circulating memory population. Future studies that evaluate shortening the interval between an ai-mAb prime and an Env boost or producing ai-mAb-Env fusion proteins to alter germinal center dynamics can improve upon an ai-mAb germline strategy, which may be warranted. These could be combined with fate mapping studies where B cells can be permanently labeled upon an initial GC entry^46,69–72^ to accurately track the history of the immunogen-responding cells.

Here, we show that the ai-mAb immunogen formulated with the SMNP adjuvant elicited a higher frequency of on-target B cells and higher titers of serum antibodies than SAS. This result is in line with the superior performance of SMNP over SAS in mice immunized with a stabilized HIV-1 Envelope trimer, where enhanced B cell germinal centers, increased T_FH_ help, and better neutralizing antibody responses were observed in mice where SMNP was used as adjuvant^60^. These results underscore the importance of adjuvant selection in the development of HIV-1 vaccines.

An important caveat of our study is that our analyses are restricted to one VRC01 class BCR with a fixed affinity, while in humans, we expect there to be a diverse pool of VRC01 class BCRs with varying affinities for either germline targeting immunogen, which could respond differently than the iGL-VRC01 BCR tested here^62,73^. Indeed, adoptive transfer studies using VRC01 class knock-in BCRs show variable B cell responses to immunization in an affinity-dependent manner^41,43,44^. Rearranging mouse models with diverse VRC01 class repertoires may serve as better small animal models for evaluating prime and prime boost regimens to elicit VRC01-class antibodies^74,75^.

We recently demonstrated that an ai-mAb immunogen could selectively target antibodies using VH3-21 and VL1-40 gene pairs that can neutralize respiratory syncytial virus^76^. Unlike VRC01-class antibodies, VH3-21/VL1-40 mAbs do not require somatic mutation to achieve potent neutralization^77^. Therefore, the use of ai-mAbs as immunogens to target VH3-21/VL1-40 B cells may still be advantageous due to their ability to rapidly elicit high-titer serum responses^32^, even if some portion of the target B cells acquire loss of function mutations in prolonged GC reactions.

The results here have implications for HIV-1 vaccine development. Our findings indicate that an ai-mAb, which is antigenically disparate from a germline-targeting Env-derived immunogen, is not an ideal immunogen to prime target B cells in a prolonged prime-boost regimen intended to drive affinity maturation of VRC01 class antibodies and that antigenic overlap between prime and boost immunogens should be a consideration in shepherding strategies.

## Methods

### Plasmids

pTT3-426c TM4Δ1-3-His (herein called 426.Mod.Core) and pTT3-426c TM4Δ1-3-His-Avi^28^ and pTT3-426c TM4Δ1-3 CD4-BS KO^78^ pTT3-eOD-GT8^32^ and pTT3 eOD-GT8-KO^32^ were previously described. A cathepsin cleavage site followed by the 2W1S peptide^32^ was incorporated at the C-terminus of the light chains of iv4 (pTT3-iv4-LC-2W1S) and iv9 (pTT3-iv9-LC-2W1S). The same peptide was added to the C-terminus of 426c.NLGS.TM4∆V1-3-HIS^28^ by replacing the C-terminal His tag with cathepsin 2W1S tag creating pTT3-426c.NLGS.TM4∆V1-3-gp120-2W1S using custom primers (IDT DNA) and the Quick Change XL kit (Agilent Cat # 200516) according to the manufacturer’s instructions.

### Protein production

pTT3-iv4-Fab-HC-SC plus pTT3-iv4-LC-2W1S, or pTT3-iv9-Fab-HC-ST plus pTT3-iv9-LC-2W1S were transfected into 293E cells at a density of 10^6^ cells ml^−1^ in Freestyle 293 media using the 293Free transfection reagent according to the manufacturer’s instructions. Expression was carried out for 6 days after which cells and cellular debris were removed by centrifugation at 4000×*g* followed by filtration through a 0.22 µm filter. Clarified cell supernatant containing iv4-SC-2W1S or iv9-ST-2W1S was passed over Ni-NTA resin (Qiagen), pre-equilibrated with Ni-NTA binding buffer (0.3 M NaCl, 20 mM Tris,10 mM imidazole, pH 8.0), followed by extensive washing with Ni-NTA binding buffer, and then eluted with 250 mM imidazole, 0.3 M NaCl, 20 mM Tris, pH 8.0 (Ni-NTA elution buffer). Proteins were further purified by SEC using an Enrich SEC 650 10 × 300 column (Bio-Rad) equilibrated in PBS.

To generate a bispecific iv4/iv9, iv4-Fab-SC-2W1S was incubated with a 1.5-fold excess of iv9-Fab-ST-2W1S for 1 h at room temperature. The complex was then separated from excess iv9-Fab ST using an Enrich SEC 650 10 × 300 column (Bio-Rad) equilibrated in PBS. iv4SC/iv9ST (referred to iv4/iv9) was either conjugated to DyLight 550-NHS ester (Thermo Fisher) and then flash frozen, or was flash frozen without a fluorescent label and stored at −20 °C.

Expression plasmids encoding Env proteins were transfected into 293F cells at a density of 10^6^ cells ml^−1^ in Freestyle 293 media (Life Technologies) using the 293Free transfection reagent (EMD Millipore) according to the manufacturer’s instructions. Expression was carried out in Freestyle 293 media for 6 days with gentle shaking at 37 °C in the presence of 5% CO_2_, after which cells and cellular debris were removed by centrifugation at 10,000×*g* followed by filtration through a 0.2 µM filter. Clarified cell supernatant containing 426c.Mod.Core.2W1S was passed over Agarose-bound Galanthus Nivalis Lectin (GNL) resin (Vector Laboratories), pre-equilibrated with 20 mM Tris, 100 mM NaCl, 1 mM EDTA, pH 7.4 (GNL binding buffer), followed by extensive washing with GNL binding buffer. Bound protein was eluted with GNL binding buffer containing 1 M methyl mannopyranoside. The eluted protein was run over a 16/60 S200 size-exclusion column (SEC) pre-equilibrated in PBS. Fractions containing 426c.Mod.Core-2W1S were pooled, aliquoted, frozen in liquid nitrogen and stored at −80 °C.

To purify recombinant proteins with a C-terminal His Tag, clarified cell supernatant was passed over Ni-NTA resin (Qiagen), pre-equilibrated with Ni-NTA binding buffer (containing 5 mM imidazole), followed by extensive washing with Ni-NTA binding buffer (supplemented with 10 mM imidazole), and then eluted with 250 mM imidazole, 0.3 M NaCl, 20 mM Tris, pH 8.0. The eluted protein was run over a 16/60 S200 size-exclusion column (SEC) pre-equilibrated in PBS. Fractions containing recombinant proteins were pooled, aliquoted, frozen in liquid nitrogen and stored at -80 °C.

### Fluorescently labelled 426 c.Mod.Core tetramer production

426c.Mod.Core-His-Avi was biotinylated with BirA biotin ligase (GeneCopoeia, Inc.) as per the manufacturer’s instructions and incubated for 1 h at RT followed by 4 °C overnight. The next morning, the protein was purified with Zeba desalting columns (Thermo Fisher Cat# A57761) according to the manufacturer’s instructions. 10 pmol of streptavidin (SA) conjugated to either BV421 (BioLegend Cat 405225) or BUV661 (BD Biosciences Cat #612979) was added per 40 pmol of biotinylated 426c.Mod.Core. The SA-fluorophore was added in five increments, with 5 min incubation on ice in the dark between each increment. Tetramers were prepared approximately 4 days before use and stored in the dark at 4 °C. Performance of tetramers was verified by staining splenocytes from naïve CD45.1^+^ WT or CD45.2^+^ glVRC01 and analyzing via flow cytometry as described below. A total of 4 pmol of 426c.Mod.Core was used per sample (2 pmol with each fluorophore).

### Mice

All mice used in our studies were housed with free access to food and water with a 12:12 light:dark cycle. The animal facilities are accredited by the Association for Assessment and Accreditation of Laboratory Animal Care. Mice were handled in accordance with the NIH Guide for the Care and Use of Laboratory Animals, and experiments were approved by the Fred Hutch Cancer Center Institutional Animal Care and Use Committee and Institutional Review Boards. Mice homozygous for the iGL-VRC01 heavy and light chains were generated by crossing iGL-VRC01 HC^79^ with iGL-VRC01 LC mice^40^. Heterozygous mice were interbred and genotyped to identify animals that were homozygous for the iGL-VRC01 heavy and light chains, which were used to set up and maintain a breeding colony as described previously^34^ (referred to as CD45.2^+^ iGL-VRC01 mice in this paper). B6.SJL-*Ptprc*^*a*^ *Pepc*^*b*^/BoyJ (herein called CD45.1) mice were purchased from Jackson Laboratory (Cat #002014) or bred in-house. Animals were euthanized by isoflurane delivered by precision vaporizer at a concentration of 1–5% until the animal demonstrates absence of a toe pinch reflex, at which point the animal is exsanguinated via cardiac puncture with a 25-gauge needle, followed by cervical dislocation.

### Adoptive transfer

Spleens were collected from female iGL-VRC01 mice aged between 7 and 12 weeks old. Single cell suspensions were produced by mechanical disruption and then filtered through a 70 µm strainer into a six-well plate containing 2 ml RBC Lysis Buffer (Thermo Fisher Cat. #A1049201). After 2 min, the samples were washed with cRPMI, and B cells were isolated using the Easy Sep mouse B cell Isolation Kit (Stem Cell Cat# 19854) according to the manufacturer’s instructions. Isolated B cells were then prepared at the desired concentration in PBS and kept on ice prior to transfer into recipient mice via retro-orbital (R.O.) injection. For proliferation studies, isolated B cells were labelled with CellTrace Violet (Thermo Fisher Cat # C34557) as per the manufacturer’s instructions. For all studies transferring <500,000 B cells, they were resuspended in Transfer Buffer (PBS + 1% heat-inactivated FBS) post B cell isolation, or CTV-labelling as appropriate. From this point on, all tubes and syringes used were pre-coated >1 h at room temperature with Transfer Buffer. The cells were also prepared at the desired concentrations in Transfer Buffer and kept on ice prior to R.O. injection of 100 µl into each recipient mouse.

### Immunizations

Male and female CD45.1^+^ mice were immunized between 6 and 16 weeks of age at the indicated time points via bilateral intra-muscular (I.M.) injection of quadriceps (50 µl per leg). They were immunized with 10 µg of either iv4/iv9 with 2W1S, or 426c.Mod.Core-2W1S. The immunogens were formulated with either 25 µg of Sigma Adjuvant System (Sigma-Aldrich Cat # S6322) or 5 µg of Saponin/Monophosphoryl Lipid A nanoparticle (SMNP)^60^ in PBS.

### Blood collection and processing

Blood was collected into Serum Gel CAT Tubes (Sarstedt Cat# 20.1344), retro-orbitally (R.O.) via cheek bleed, or via cardiac puncture (at endpoint). Serum was separated from whole blood via centrifugation and then heat-inactivated at 56 °C for 10–60 min and stored at 4 °C until use.

To purify IgG for antibody transfer, blood was collected via terminal cardiac puncture from donor mice and processed as detailed above. The serum was diluted 1:1 with protein G IgG binding buffer (Thermo Fisher Scientific Cat# 21019), then passed over a column containing protein G agarose (Thermo Fisher Scientific Cat# 20397). The column was washed with 10× the column volume of PBS before eluting with IgG elution buffer. The eluate was then buffer exchanged into PBS and concentrated, diluted to 2.5 mg/ml and passed through a 0.2 µm filter. 500 µg of purified IgG per mouse was delivered in a 200 µl volume via R.O. injection approximately five hours before immunization.

### Flow cytometry

Inguinal, axillary, and lumbar lymph nodes were collected, pooled, and the spleens were harvested in 1 ml cRPMI at necropsy. Spleens were processed into single-cell suspensions as described above. Each sample was then resuspended in 500 µl of FACS Buffer and enriched for B cells using the Easy Sep mouse B cell Isolation Kit (Stem Cell Cat# 19854). After isolation, each spleen sample was resuspended in 1 ml FACS Buffer. Lymph nodes were mechanically disrupted in cRPMI. The resulting suspension was then transferred to a 96-well 30–40 µm filter plate (Cytiva Cat#8227), along with the B-cell-enriched splenocytes. All samples were then resuspended in FACS buffer (250 µl for lymph nodes or 750 µl for spleens). Total lymph nodes and one-third of each spleen were stained for flow cytometry. Samples were first stained with 4 pmol of 426c.Mod.core tetramers (2 pmol conjugated to 0.5 mol of SA-BV421 or SA-BUV661 as described above) in FACS buffer for 30 min in the dark on ice. Samples were then washed once with FACS buffer and stained for surface markers with the following cocktail in FACS buffer: anti-GL7 PerCP-Cy5.5 (1:200 dilution, Biolegend, Cat# 144609), anti-CD45.2 Alexa Fluor 647 (1:100 dilution, BioLegend, Cat# 109818), anti-CD38 Alexa Fluor 700 (1:100 dilution, eBioscience, Cat# 56-0381-82), anti-CD3 BV711 (1:100 dilution, BioLegend, Cat# 100349), anti-Ly6G BV711 (1:100 dilution, BioLegend, Cat# 127643), anti-F4/80 BV711 (1:100 dilution, BioLegend, Cat# 123147), anti-CD45R/B220 BV786 (1:250 dilution, BD Biosciences, Cat# 563894), anti-CD45.1 PE-eFluor 610 (1:100 dilution, eBioscience, Cat# 61-0453-82), anti-CD138 PE-Cy7 (1:100 dilution, BioLegend, Cat# 142514), anti-CD19 BUV395 (1:200 dilution, BD Biosciences, Cat# 563557), CD16/32 Fc block (1:200 dilution, BioLegend, Cat# 101302),, and Fixable Viability Dye eFluor 506 (1:200 dilution, eBioscience, Cat# 65-0866-14). After 30 min on ice in the dark, the samples were washed once with FACS buffer before being fixed and permeabilized in Foxp3/Transcription factor Fix and Perm buffer (eBioscience, Cat# 00-5523-00) for 1 h on ice in the dark. They were then washed once with permeabilization buffer and intracellularly stained with Bcl6-FITC (1:100 dilution, BioLegend, Cat# 358513) in permeabilization buffer overnight at 4 °C in the dark. The next morning, all samples were washed once with FACS buffer. They were then resuspended in 200 µl FACS buffer with 20,000 AccuCheck counting beads (Thermo Fisher Scientific Cat# PCB100) and kept on ice in the dark until acquisition on the flow cytometer (BD FACSymphony A5 with BD Diva software) the same day. Flow cytometry data were analyzed with FlowJo (10.8.1) with the following gating strategies. For proliferation studies: lymphocytes/single cells/live, CD3^−^, Ly6G^−^, F4/80^−^/CD19^+^ B220^+^ (B cells)/CD45.2^+^/CTV^+^ or CTV^−^. For prevalence of CD45.2^+^ cells as a percentage of total B cells: lymphocytes/single cells/live, CD3^−^, Ly6G^−^, F4/80^−^/CD19^+^ B220^+^ (B cells)/CD45.2^+^ (iGL-VRC01). For prevalence of iGL-VRC01 cells as a percentage of B cells in the GC: lymphocytes/single cells/live, CD3^−^, Ly6G^−^, F4/80^−^/CD19^+^ B220^+^ (B cells)/Bcl-6^+^ GL-7^+^ (GC)/CD45.2^+^. For 426c.Mod.core^+^ cells as percentage of total B cells: lymphocytes/single cells/live, CD3^−^, Ly6G^−^, F4/80^−^/CD19^+^, B220^+^ (B cells)/426c.Mod.core-BV421^+^, 426c.Mod.core-BUV661^+^/CD45.1^+^ or CD45.2^+^ (as a percentage of B cells). For composition of 426c.Mod.core^+^ cells as percentage of B cells in the GC: lymphocytes/single cells/live, CD3^−^, Ly6G^−^, F4/80^−^/CD19^+^, B220^+^ (B cells)/Bcl-6^+^, GL-7^+^(GC)/426c.Mod.core-BV421^+^, 426c.Mod.core^+^-BUV661+/CD45.1^+^ or CD45.2^+^ (as a percentage of GC cells). Limits of detection (LoD) were determined by staining 6 naïve CD45.1^+^ WT mice, which did not receive any CD45.2^+^ cells, with the same panel as the experimental samples. The average frequency of cells that show spurious CD45.2^+^/CD45.1^−^ staining in these control mice was set as the LoD. Similarly, 4 CD45.1^+^ WT mice which did not receive any CD45.2^+^ cells were stained with 426c.Mod.core. The average frequency of cells that showed spurious 426c.Mod.core staining in these control mice was set as the LoD.

None of the control mice had CD45.2^+^ splenic GC B cells. Therefore, the theoretical LoD of this population was obtained by calculating the fold difference between the percentage of total B cells and of GC B cells that were CD45.2^+^ in the lymph nodes. This fold difference was then multiplied by the percentage splenic B cells that were CD45.2^+^ to obtain an estimated LoD for CD45.2^+^ cells in splenic GCs. An estimated LoD of 426c.Mod.core^+^ cells in splenic GCs that are CD45.2^+^ were calculated the same way. This 426c.Mod.core^+^ CD45.2^+^ LoD was combined with the observed 426 c.Mod.core^+^ CD45.1^+^ LoD to determine the LoD for the total 426c.Mod.core^+^ in splenic GCs. For the purposes of quantification and visualization, any values obtained from experimental samples that were below the LoD were assigned a value equal to the LoD.

### Single B cell sorting and sequencing

Lymph node samples from mice were prepared as described above and stained for surface markers with the following cocktail in FACS buffer: anti-CD45.2 Alexa Fluor 647 (1:100 dilution, BioLegend, Cat# 109818), anti-CD38 Alexa Fluor 700 (1:100 dilution, eBioscience, Cat# 56-0381-82), anti-CD3 BV711 (1:100 dilution, BioLegend, Cat# 100349), anti-Ly6G BV711 (1:100 dilution, BioLegend, Cat# 127643), anti-F4/80 BV711 (1:100 dilution, BioLegend, Cat# 123147), anti-mouse Kappa FITC (1:100, SouthernBiotech, Cat# 1170-02), anti-CD95 BUV737 (1:200, BD Biosciences, Cat# 741763), anti-CD45R/B220 PerCP (1:250 dilution, BioLegend, Cat# 103234), anti-CD45.1 PE-eFluor 610 (1:100 dilution, eBioscience, Cat# 61-0453-82), anti-CD19 BUV395 (1:200 dilution, BD Biosciences, Cat# 563557), CD16/32 Fc block (1:200 dilution, BioLegend, Cat# 101302), and Fixable Viability Dye eFluor 506 (1:200 dilution, eBioscience, Cat# 65-0866-14). Cells were kept at 4 °C in the dark prior to running on a BD FACSymphony S6 using BD Diva software. iGL-VRC01 GC^+^ B cells were identified as lymphocytes/single cells/live/CD3^−^, Ly6G^−^, F4/80^−^/CD19^+^, B220^+^/CD45.2^+^, CD45.1^−^/CD95^+^, CD38^−^. CD45^+^GC B cells were single-cell sorted into individual wells of 96-well plates and frozen on dry ice.

To sequence the VH and VL transcripts from single sorted B cells, cDNA was generated with Superscript IV (Invitrogen Cat# 18090010), and then the VH and VL sequences were recovered using gene-specific primers and cycling conditions as previously described^40,80^. VH and VL amplicons were Sanger sequenced (Genewiz) and analyzed using Geneious Prime (Dotmatics).

### Measurement of serum antibody titers by ELISA

Serum titers to eOD-GT8, 426c.Mod.Core (without 2W1S) and their respective KO versions were determined via ELISA. Clear flat-bottom 384-well plates (Thermo Scientific Cat. #464718) were coated with 30 µl of coating buffer (0.1 M sodium bicarbonate) containing 2 µg/ml of target protein at room temperature overnight. The next morning, the plate was washed and blocked with blocking (PBS + 10% non-fat milk + 0.03% Tween20) for 90 min. Serum serially diluted in blocking buffer was added to the plate in duplicate. For each target protein, 16 control wells were incubated with blocking buffer without serum. Plates were incubated for 1 h at 37 °C and then washed again. The secondary antibody, goat anti-mouse IgG-HRP (BioLegend Cat # 405306) was diluted 1:3000 in blocking buffer and added to each well, followed by a final 1 h incubation at 37 °C. The plates were washed a final time and were developed using 30 µl SureBlue Reserve TMB peroxidase substrate (SeraCare KPL, Cat# 5120-0080) per well for five minutes at room temperature. After this time, 30 µl of 1 N H_2_SO_4_ was added to each well to stop the reaction, and the OD_450_ of the plates was read using a SpectraMax i3x (Molecular Devices) with SoftMax Pro 6.5.1 software. All wash steps consisted of four cycles of 120 µl of wash buffer (PBS + 0.02% Tween20) using a BioTek 405 TS microplate washer. Each sample’s titers to both the target protein and corresponding KO were measured on the same plate to enable more accurate comparisons. Similarly, where serum from multiple time points from the same mouse was assessed, they were also on the same plate. The average OD_450_ for the negative control wells was subtracted from the averaged values for the duplicate wells of each sample. The OD cut-off value for each protein was determined as the average OD_450_ for the negative control wells + 3 × standard deviations. Endpoint titers were determined by performing a sigmoidal dose–response (variable slope) nonlinear regression analysis with interpolated *x* values. Where endpoint titers could not be determined, 1:5 was used (equivalent to half the lowest dilution tested).

### Statistical analysis

GraphPad Prism (version 10) was used to perform Mann–Whitney and Kruskal–Wallis tests. *P* values < 0.05 were regarded as significant and displayed.

## Supplementary information

Supplementary Figures 6.26.25

## Data Availability

Sequences of produced mAbs (MLH_1–16) were deposited in GenBank under accession numbers PV636323–PV636354. All other data included in this study will be made available upon request to amcguire@fredhutch.org.
