# Supplementary material for: Priming VRC01-precursor B cells with non-envelope immunogens disfavors boosting with HIV-1 envelope
Source: NPJ Vaccines. 2025 Aug 5;10:185. doi: 10.1038/s41541-025-01235-5 (PMC12325944; doi:10.1038/s41541-025-01235-5)
Supplement: Supplementary file 1 — Supplementary Figures 6.26.25 [file 41541_2025_1235_MOESM1_ESM.pdf]

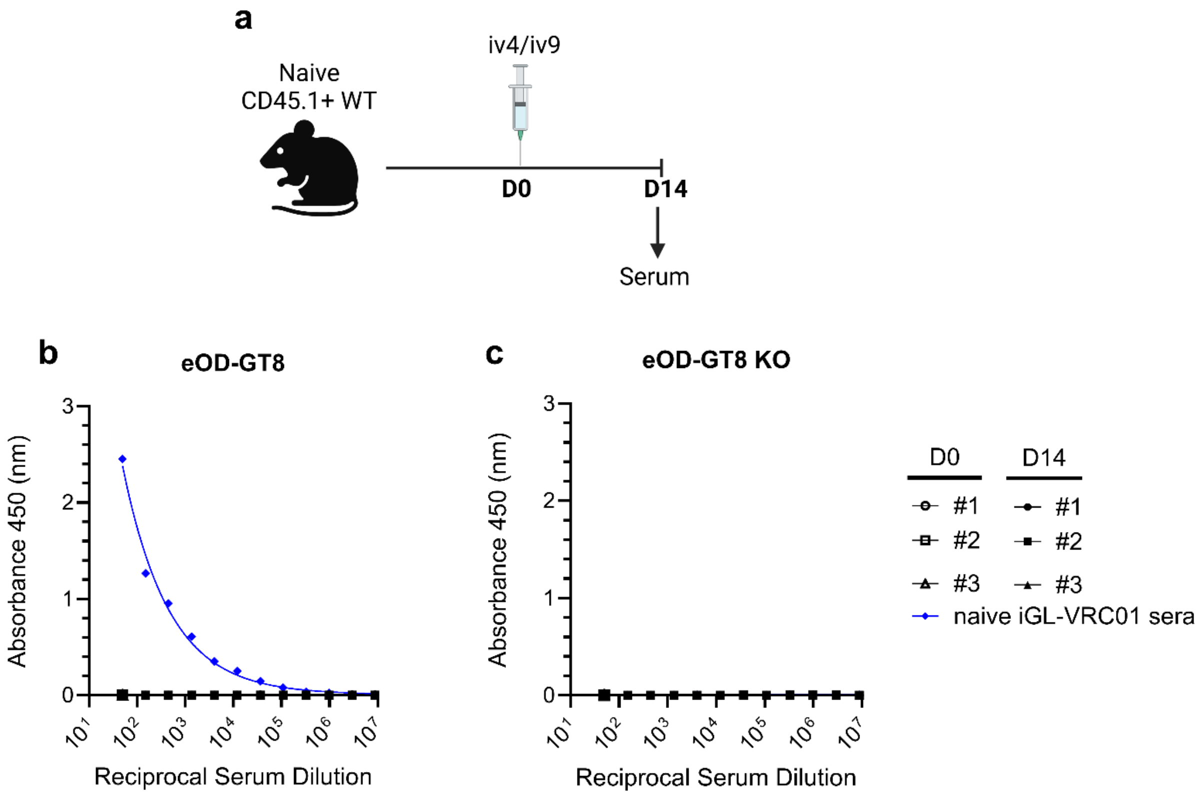

Supplementary Figure 1. iv4/iv9 does not elicit eOD-GT8 reactive antibodies. Related to Figure 1. **a** Schematic of experiment. CD45.1^{+} mice (n=3) were immunized iv4/iv9-2W1S and serum was collected 14 days later. Created with BioRender. **b-c** Serum binding titers to eOD-GT8 (**b**) and eOD-GT8 KO (**c**) were measured by ELISA. Naïve sera from an iGL-VRC01 knock-in mouse was included as a control (blue) in **b** and **c**.

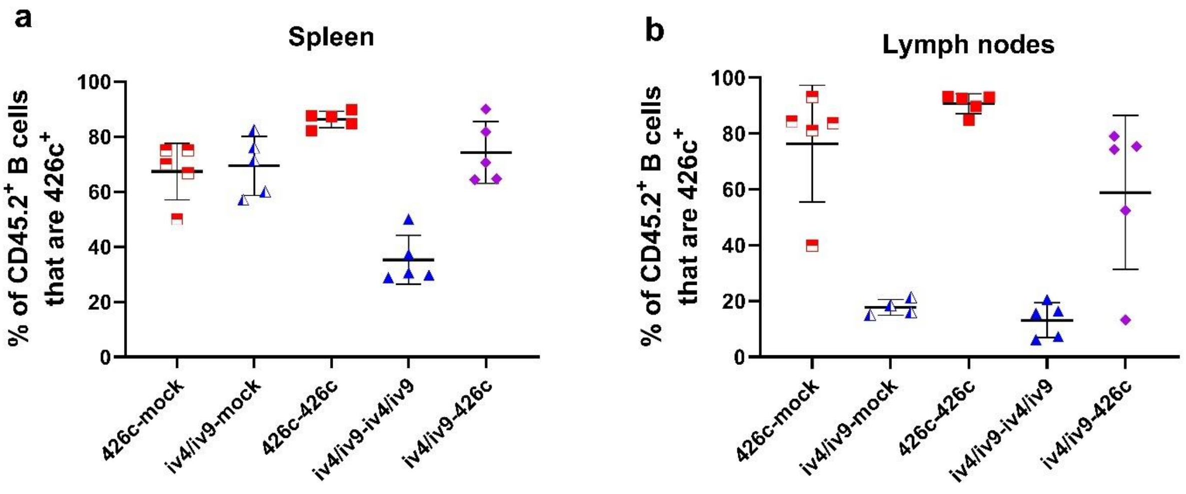

Supplementary Figure 2. Frequency of CD45.2+ B cells that bind to 426c.Mod.Core. Related to Figures 2-3. **a-b** The percentage of CD45.2^{+} B cells from the indicated groups shown in Figure 2 that stained positive with fluorescently labeled 426c.Mod.core in the spleen (**a**) and lymph nodes (**b**) are shown as indicated.

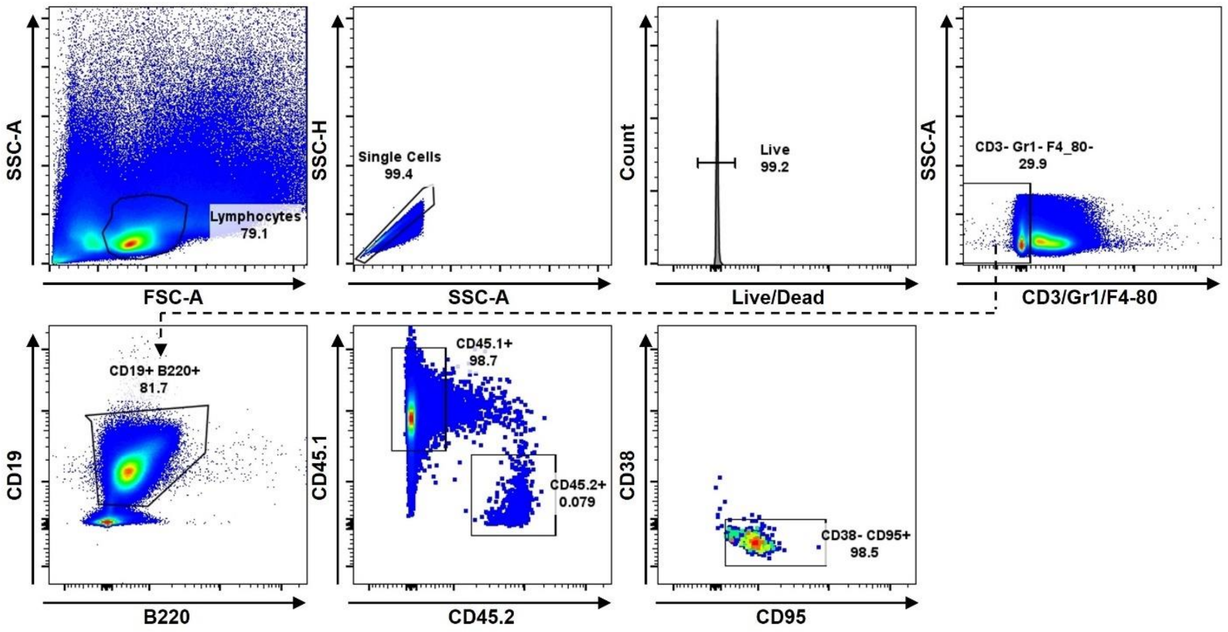

Supplementary Figure 3. Gating strategy to identify and sort CD45.2+ GC cells from the lymph nodes of iv4/iv9 immunized mice. Related to Figure 3. Gating as follows: Lymphocytes → Single cells → Live cells → $CD3^-$, $Gr1^-$, $F4\text{-}80^-$ → $CD19^+$, $B220^+$ → $CD45.1^-$, $CD45.2^+$ → $CD38^-$, $CD95^+$. Percentages of parent gate indicated, representative gating from the D21 sort described in Fig. 3.

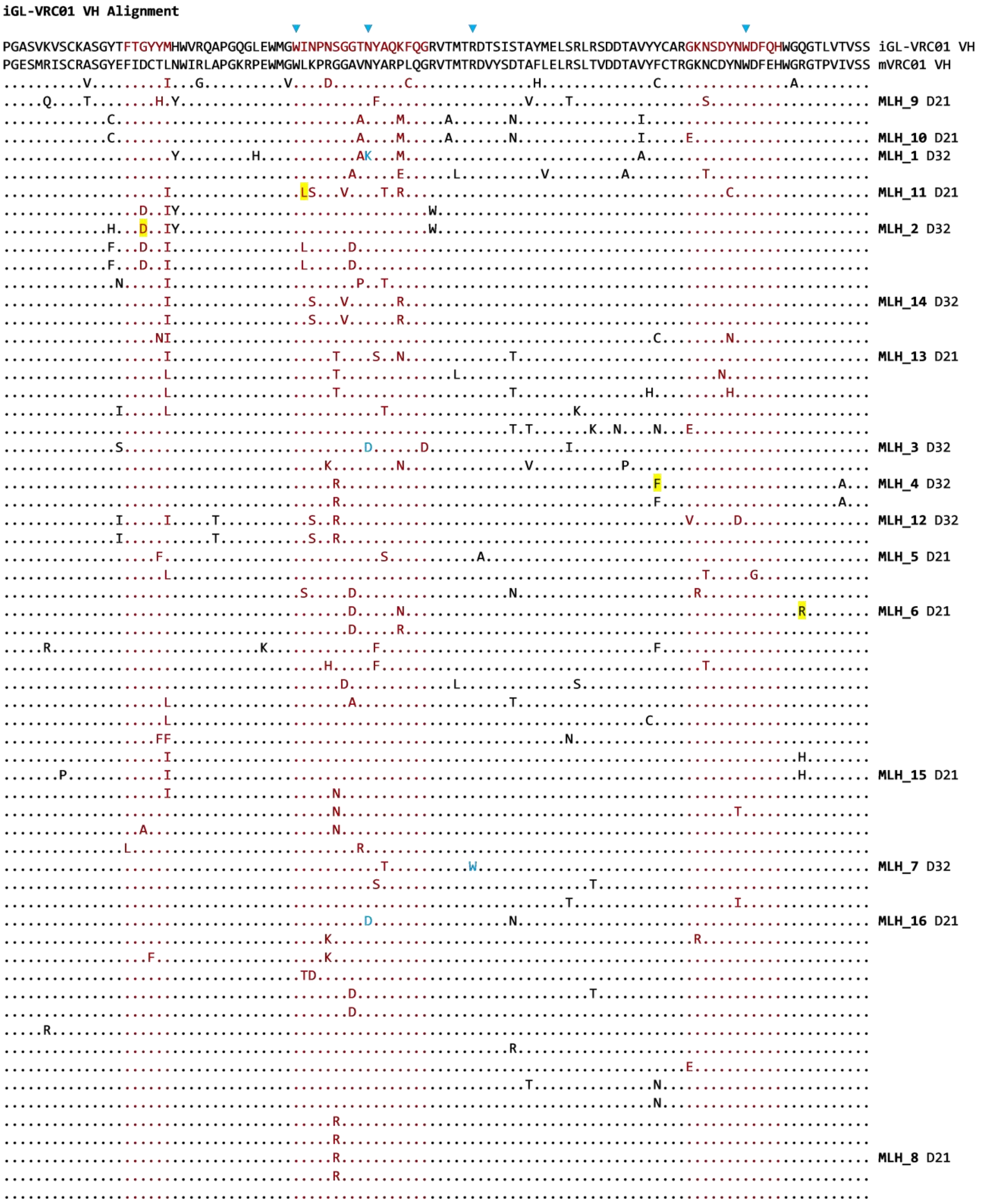

Supplementary Figure 4a. Alignment of CD45.2+ BCR heavy chain transcripts. Related to Figure 3. Sequences of VH transcripts recovered from single-cell sorted CD45.2+ LN GC B cells aligned to AA#s 14-121 of iGL-VRC01 VH (top row) and mature

VRC01 VH (mVRC01 VH, second row). Dots represent amino acids that are identical to iGL-VRC01 VH. Letters represent animo acid substitutions. Amino acids in CDR regions are colored red. Signature VRC01-class like (PVL) amino acids, W50, N58, R71, and W100B, are indicated by blue arrows, and mutated PVL residues are colored in blue. Heavy chains corresponding to mAbs selected for recombinant production are labeled on right along with their isolated timepoint, and within these, mutated residues that are identical to mature VRC01 are highlighted in yellow.

iGL-VRC01 VL Alignment

```
TLSCRASQSVSSYLAWYQQKPGQAPRLLIYDASNRATGIPARFSGSGSGTDFTLTISSLEPEDFAVYYCQQYEFFGQGTKLEIK iGL-VRC01 VL
IISCRTSQYGS--LAWYQQRPGQAPRLVIYSGSTRAAGIPDRFSGSRWGPDYNLTISNLESGDFGVYYCQQYEFFGQGTKVDIK mVRC01 VL
......R....N..............V......I......G...........................W...............
..........................................V.........IFI............................. MLH_6 D21
.....T...I...............W..........................................................
........TI............R.............................................................
..............D............................R........................................
..................K..............R.T................................................
......................................................................K..........L.. MLH_14 D32
.................L............G..................................................... MLH_5 D21
.............S........L......H......................................................
..........N...........L.............................................................
..........N.............................................................G...........
.................................................A................F.................
...............................T....................................................
...............................T.................................................... MLH_15 D21
.............................C.T.................................................L..
...............................T.................................................L.. MLH_12 D32
....................................I............................................L..
.................................................................................L..
.................................................................................L..
.................................................................................L.. MLH_3 D32
..............D............................R........................................
................H...........F.......................................................
.........................................S........................C.................
.......................................................................H............
......................L............................................................. MLH_8 D21
................................................................T...................
.............................N......................................................
.............................N......................................................
.............................N...................................................... MLH_16 D21
......................................V.............................................
..........................................V.........................................
.....................................................................H..............
..................................................................S.................
............S.......................................................................
...........................V........................................................
.................................D..................................................
.................H..................................................................
...............................V....................................................
.......L...............................................................F............ MLH_7 D32
.......................................................................F............ MLH_11 D21
.......................................................................F............
.................................................................................... MLH_4 D32
.................................................................................... MLH_1 D32
....................................................................................
....................................................................................
....................................................................................
....................................................................................
.................................................................................... MLH_2 D32
....................................................................................
....................................................................................
....................................................................................
....................................................................................
....................................................................................
....................................................................................
.................................................................................... MLH_10 D21
....................................................................................
....................................................................................
.................................................................................... MLH_9 D21
....................................................................................
....................................................................................
....................................................................................
.................................................................................... MLH_13 D21
```

Supplementary Figure 4b. Alignment of CD45.2+ BCR Light chain transcripts. Related to Figure 3.Sequences of VL transcripts recovered from single-cell sorted CD45.2+ LN GC B cells from iv4/iv9 immunized mice aligned to AA#s 20-103 iGL-VRC01 VL (top row) and mature VRC01 LC (mVRC01 LC, second row). Dots represent amino acids that are identical to iGL-VRC01. Letters represent animo acid substitutions. Amino acids in CDR regions are colored red. Representative mAbs selected for recombinant production are labeled on right along with their isolation timepoint.

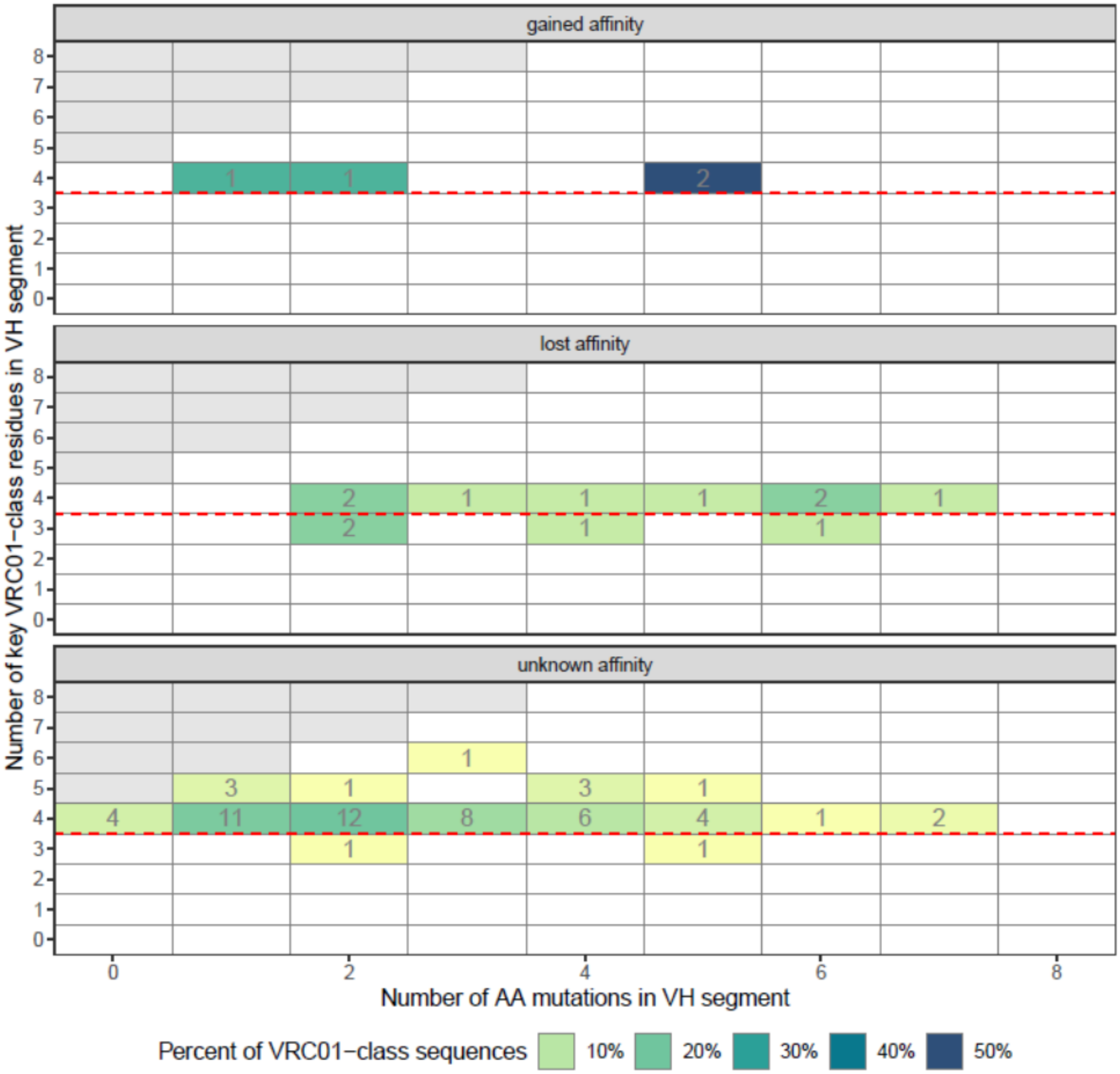

Supplementary Figure 5. Analysis of key VRC01-class contacts. Related to Figure 3. Number of key VRC01-class residues vs. total number of amino acid mutations in the VH segment of transcripts recovered from single-cell sorted CD45.2$^+$ LN GC B cells from iv4/iv9 immunized mice. VH sequences from mAbs that gained affinity (top), lost affinity (middle), or were not reconstructed as mAbs (bottom) are plotted separately. The rectangular tiles are colored by the percentage of VRC01-class sequences (within each category) and labeled by the count of VRC01-class sequences. Rectangular tiles that are gray represent unreachable states (number of key VRC01-class mutations greater than total number of mutations). The set of key VRC01-class residues[1,2] is shown in Supplementary Table 2. Entries below the dashed red line represent sequences that have fewer key VRC01-class residues than the germline VH1-2*02

sequence (i.e., less than four key residues). One ’unknown affinity’ sequence with poor quality IMGT alignment, which resulted in a high number of inferred mutations, has been filtered out for the purpose of this analysis.

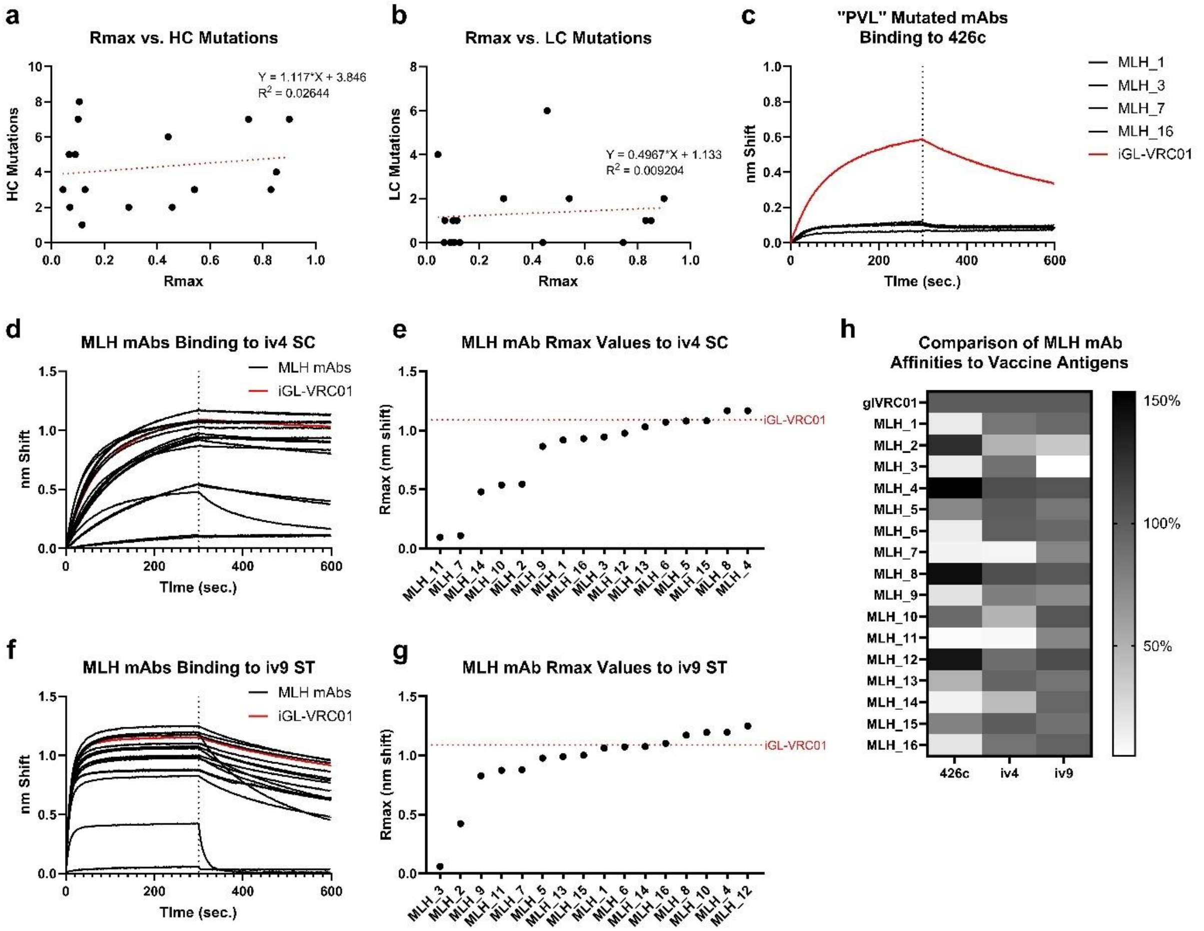

Supplementary Figure 6. Correlation analysis of VH and VL mutations relative to R_{max} of recombinant mAb binding to 426c.Mod.Core,"PVL" mutated mAb BLI traces and BLI traces of MLH mAbs to iv4 SC and iv9 ST. Related to Figure 3. **a-b** Linear regression analysis of R_{max} values from Fig. 3i vs. number of heavy chain mutations (**a**) or light chain mutations (**b**) relative to iGL-VRC01. **c** Representative BLI traces of "PVL" mutated mAbs (black) compared to iGL-VRC01 IgG (red). **d** Representative BLI traces of MLH mAbs compared to iGL-VRC01 against iv4 SpyCatcher (SC). **e** R_{max} values of sorted mAbs to iv4 SC. R_{max} of iGL-VRC01 to iv4 SC shown by red dotted line. **f** Representative BLI traces of MLH mAbs compared to iGL-VRC01 against iv9 SpyTag (ST). **g** R_{max} values of sorted mAbs to iv9 ST (SC). R_{max} of iGL-VRC01 to iv9 SC shown by red dotted line. **h** Heat map of R_{max} values of MLH mAbs to vaccine antigens as a percentage of iGL-VRC01 R_{max} to the same antigens.

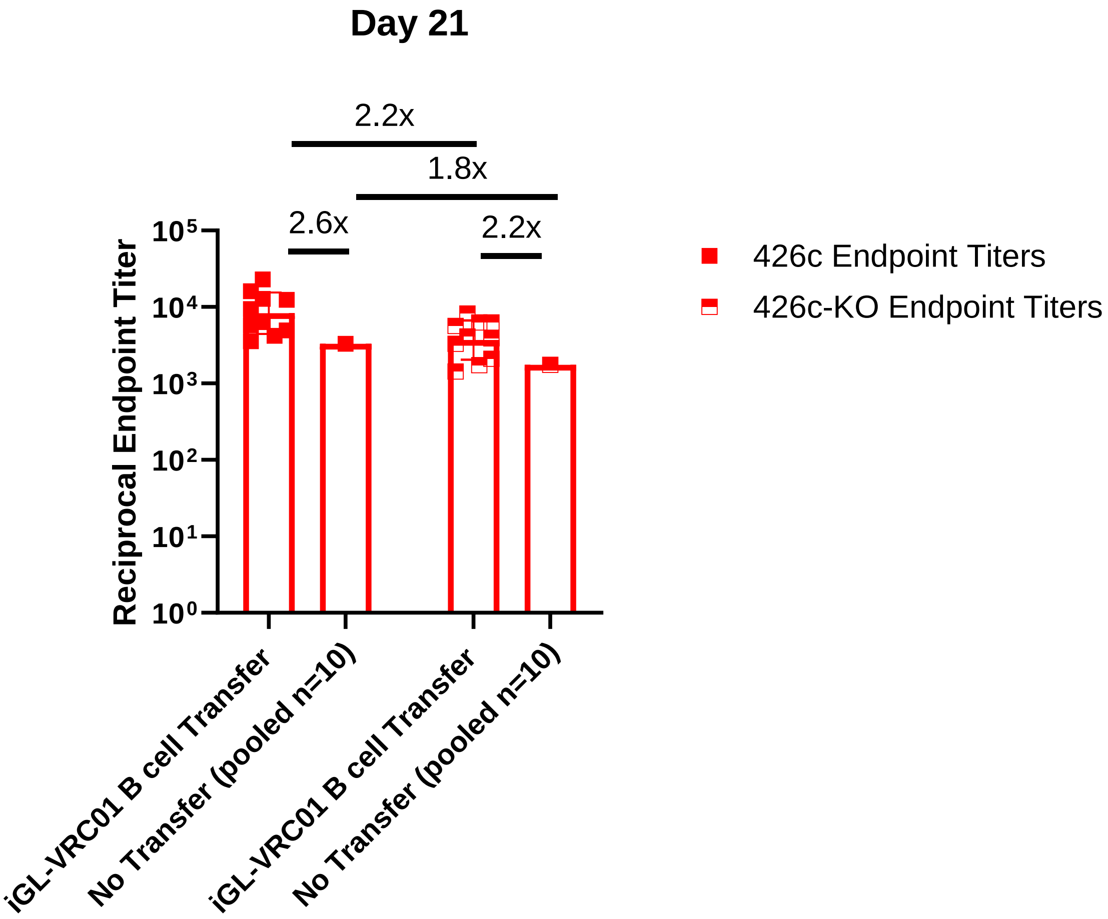

Supplementary Figure 7. Serum binding titers to 426c.Mod.Core in immunized mice with and without iGL-VRC01 B cell transfer. Related to Figures 4 and 7. CD45.1^{+} mice which did not receive transfer of iGL-VRC01 CD45.2^{+} B cells (No Transfer) from Figure 7, were immunized with 426c.Mod.Core (n=10). 21 days later the serum was collected and pooled. The endpoint binding titer of pooled sera to 426c.Mod.Core (426c) or 426c.Mod.Core knockout (426c-KO) was measured by ELISA. The day 21 endpoint titers from CD45.1^{+} mice that received 5000 iGL-VRC01 CD45.2^{+} B cells prior to immunization from Figure 3 are shown for comparison (iGL-VRC01 B Cell Transfer). The fold-differences between the groups are shown above the bars.

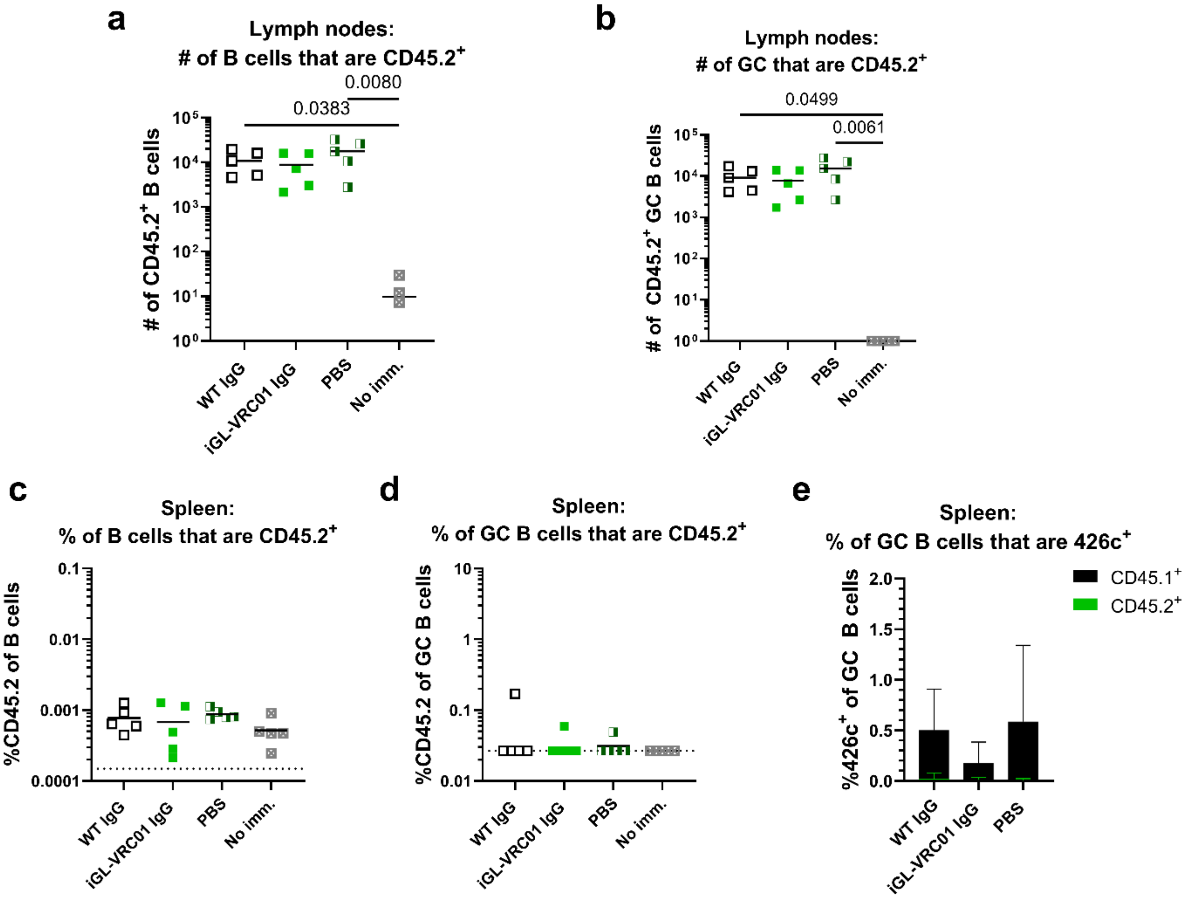

Supplementary Figure 8. Effect of on-target antibodies on B cell responses to a 426c.Mod.Core prime in the lymph nodes and spleen. Related to Figure 5. **a-b** Number of B cells that are CD45.2^{+} (**a**) and number of GC B cells that are CD45.2^{+} in the lymph nodes (**b**) of the mice from Figure 5. P values are reported as determined by Kruskal-Wallis test with Dunn's multiple comparisons. Splenocytes were harvested from the mice in Figure 5. **c** frequency of total B cells that are CD45.2^{+} in the spleen at day 10. For mice where no cells were observed they are reported as 1 for graphical purposes. **d** Frequency of germinal (GC) B cells that are CD45.2^{+} in the spleen at day 10. Each data point represents one mouse and the mean is indicated by a bar. **e** Mean percentage of 426c.Mod.Core^{+} GC B cells in the spleen at day 10. The height of the bar indicates the total frequency of 426c.Mod.Core^{+} cells, and the colors indicate whether they are of CD45.1^{+} (black) or CD45.2^{+} (green) origin. Bars represent the mean and error bars represent the standard deviation of n=5 mice per group.

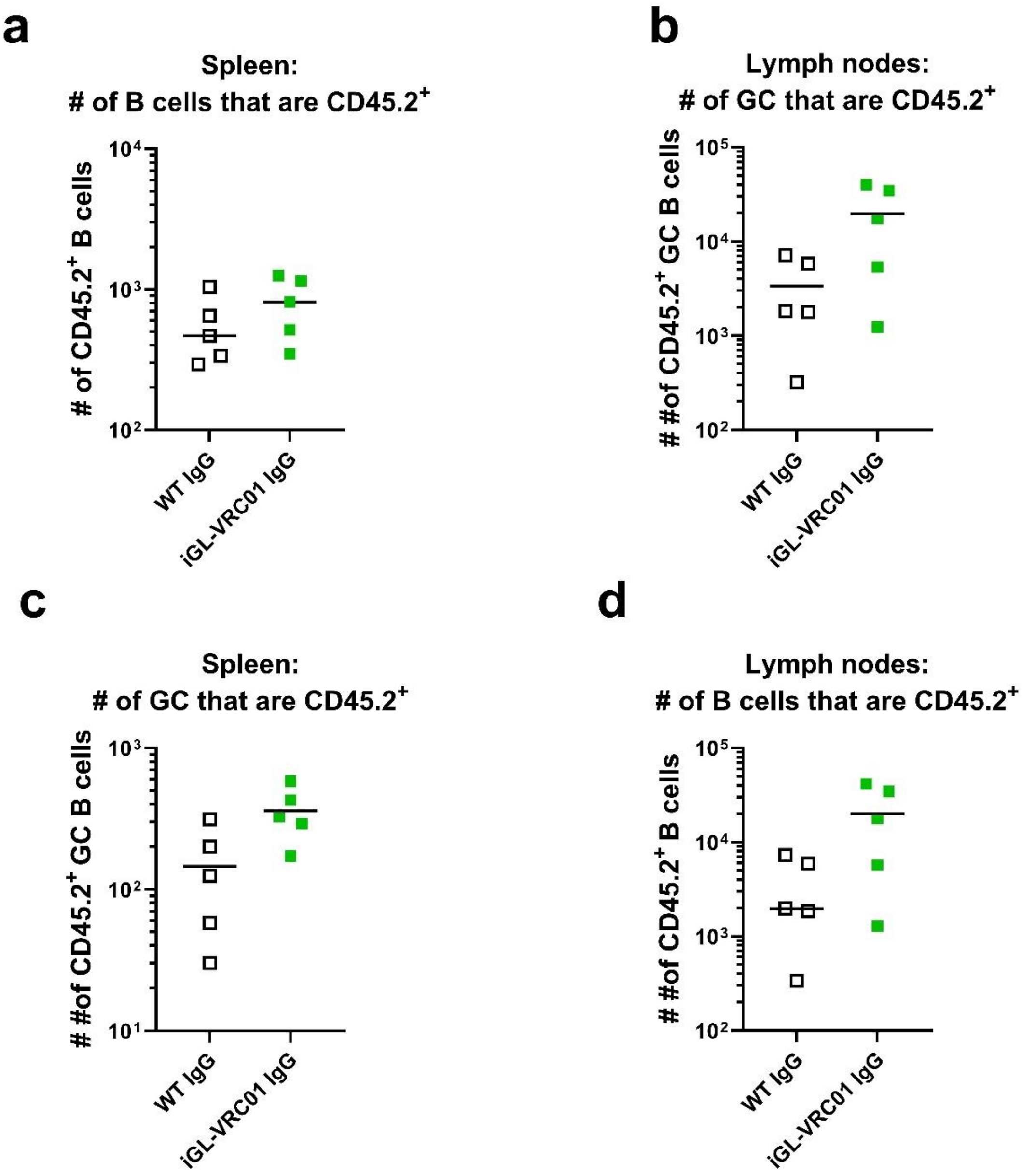

Supplementary Figure 9. Effect of on-target antibody transfer on response to boost. Related to Figure 6. **a-b** Number of CD45.2$^+$ B cells in the spleen (**a**) and lymph nodes (**b**) from the animals in Figure 6. **c-d** Number of CD45.2$^+$ GC B cells in the spleen (**c**) and lymph nodes (**d**) from the animals in Figure 6. Each data point represents one mouse (n=5) and the bar indicates the mean.

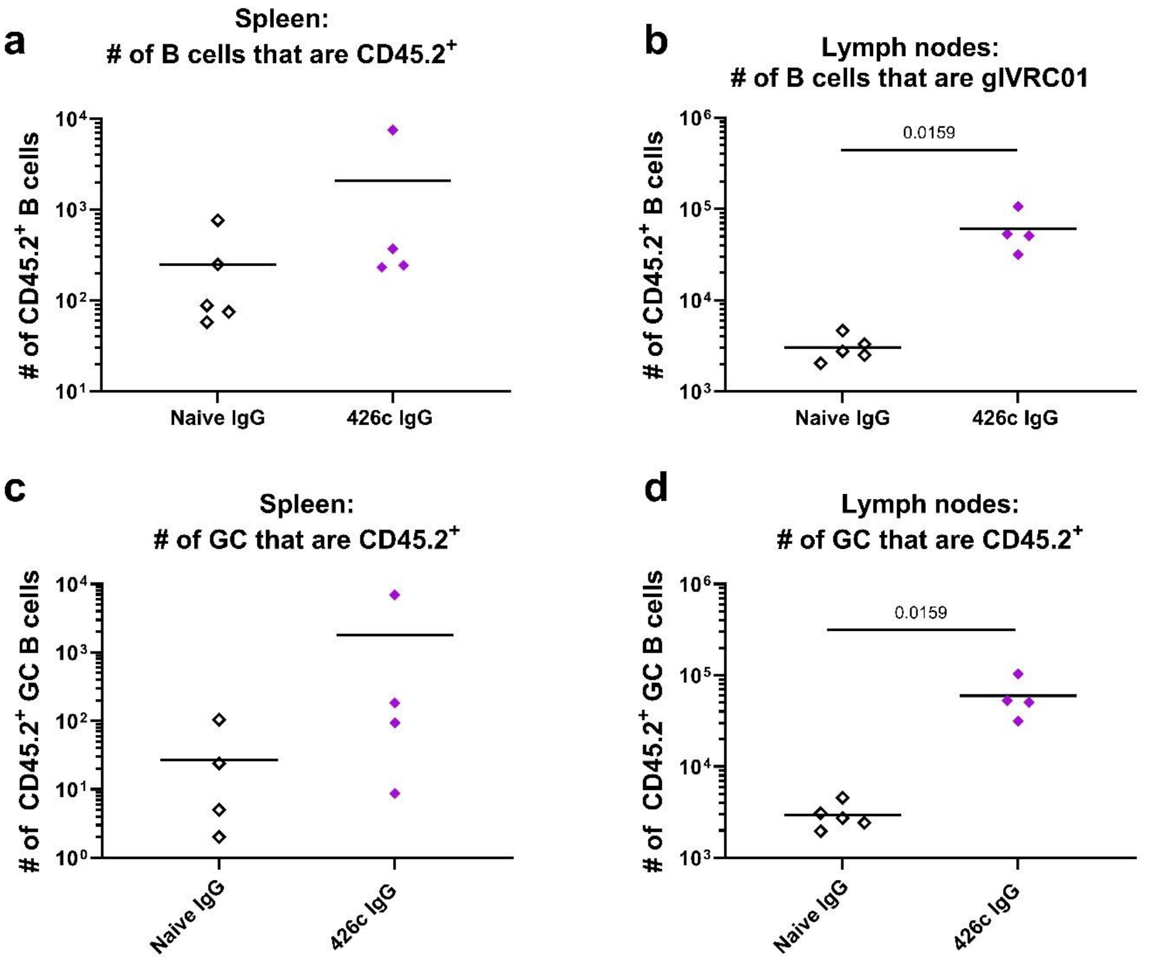

Supplementary Figure 10. Effect of off-target antibody transfer on response to boost. Related to Figure 7. **a-b** Number of CD45.2$^+$ B cells in the spleen (**a**) and lymph nodes (**b**) from the animals in Figure 7. **c-d** Number of CD45.2$^+$ GC B cells in the spleen (**c**) and lymph nodes (**d**) from the animals in Figure 7. Each data point represents one mouse and the bar indicates the mean. P values determined by Mann-Whitney tests.

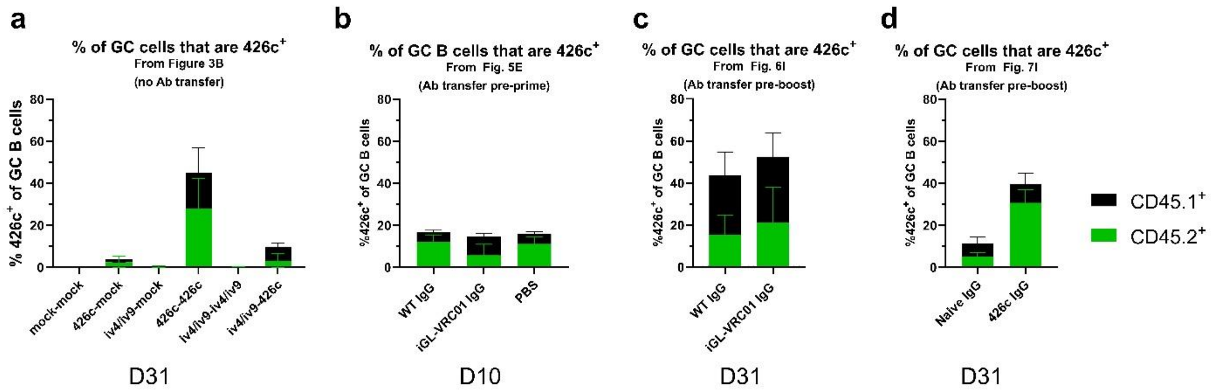

Supplementary Figure 11. Mean percentage of 426c.Mod.Core^{+} GC B cells in the lymph nodes across several experiments. Related to Figures 3, 5, 6 and 7. The height of the bar indicates the total frequency of 426c.Mod.Core^{+} cells, and the colors indicate whether they are of CD45.1^{+} (black) or CD45.2^{+} (green) origin. Bars represent the mean and error bars represent the standard deviation. Data is reproduced from Figure 3b (**a**), 5e (**b**) Figure 6i (**c**) and Figure 7i (**d**) and plotted on the same scale for comparison.

Supplementary Table 1. GenBank accession numbers for produced mAbs.

mAb	GenBank Accession Number
MLH_1-VH	PV636323
MLH_1-VL	PV636324
MLH_2-VH	PV636325
MLH_2-VL	PV636326
MLH_3-VH	PV636327
MLH_3-VL	PV636328
MLH_4-VH	PV636329
MLH_4-VL	PV636330
MLH_5-VH	PV636331
MLH_5-VL	PV636332
MLH_6-VH	PV636333
MLH_6-VL	PV636334
MLH_7-VH	PV636335
MLH_7-VL	PV636336
MLH_8-VH	PV636337
MLH_8-VL	PV636338
MLH_9-VH	PV636339
MLH_9-VL	PV636340
MLH_10-VH	PV636341
MLH_10-VL	PV636342
MLH_11-VH	PV636343
MLH_11-VL	PV636344
MLH_12-VH	PV636345
MLH_12-VL	PV636346
MLH_13-VH	PV636347
MLH_13-VL	PV636348
MLH_14-VH	PV636349
MLH_14-VL	PV636350
MLH_15-VH	PV636351
MLH_15-VL	PV636352
MLH_16-VH	PV636353
MLH_16-VL	PV636354

Supplementary Table 2: Key VRC01-class germline residues and mutations in the VH segment, including four germline-encoded residues and nineteen mutated positions[1,2].

Position (Kabat)	19	31	32	33	34	35	37	50	52	53	54	56	57	58	61	62	66	71	73	74	75	76	77
VH1-2*02	K	G	Y	Y	M	H	V	W	N	N	S	G	T	N	Q	K	R	R	T	S	I	S	T
VRC01-class	R	D / N	C	I / T	L	N	I / W	L / W	K	R / V	F / G	A	V	N	R	P / Q	R	R	I / V	Y	R / S	D / E	I

Supplementary Table 3: Key residues in mAbs that gained affinity

Position	19	31	32	33	34	35	37	50	52	53	54	56	57	58	61	62	66	71	73	74	75	76	77
MLH_2	K	D	Y	Y	I	Y	V	W	N	N	S	G	T	N	Q	K	W	R	T	S	I	S	T
MLH_4	K	G	Y	Y	M	H	V	W	N	N	R	G	T	N	Q	K	R	R	T	S	I	S	T
MLH_8	K	G	Y	Y	M	H	V	W	N	N	R	G	T	N	Q	K	R	R	T	S	I	S	T
MLH_12	K	G	Y	Y	I	H	V	W	S	N	R	G	T	N	Q	K	R	R	T	S	I	S	T

Red = off-track mutation
Blue = on-track mutation

Supplementary Table 4: Key residues in mAbs that lost affinity

Position	19	31	32	33	34	35	37	50	52	53	54	56	57	58	61	62	66	71	73	74	75	76	77
MLH_1	K	G	Y	Y	M	Y	V	W	N	N	S	G	A	K	Q	M	R	R	T	S	I	S	T
MLH_3	K	G	Y	Y	M	H	V	W	N	N	S	G	T	D	Q	K	R	R	T	S	I	S	T
MLH_5	K	G	Y	F	M	H	V	W	N	N	S	G	T	N	Q	K	R	R	T	S	I	S	T
MLH_6	K	G	Y	Y	M	H	V	W	N	N	S	D	T	N	Q	N	R	R	T	S	I	S	T
MLH_7	K	G	Y	Y	M	H	V	W	N	N	S	G	T	N	Q	K	R	W	T	S	I	S	T
MLH_9	Q	G	Y	H	M	Y	V	W	N	N	S	G	T	N	Q	K	R	R	T	S	I	S	T
MLH_10	K	G	Y	Y	M	H	V	W	N	N	S	G	A	N	Q	M	R	R	T	S	I	N	T
MLH_11	K	G	Y	Y	I	H	V	W	S	N	S	G	T	N	Q	R	R	R	T	S	I	S	T
MLH_13	K	G	Y	Y	I	H	V	W	N	N	T	G	T	N	Q	N	R	R	T	S	I	T	T
MLH_14	K	G	Y	Y	I	H	V	W	S	N	S	G	T	N	Q	R	R	R	T	S	I	S	T
MLH_15	K	G	Y	Y	I	H	V	W	N	N	S	G	T	N	Q	K	R	R	T	S	I	S	T
MLH_16	K	G	Y	Y	M	H	V	W	N	N	S	G	T	D	Q	K	R	R	T	S	I	N	T

Red = off-track mutation
Blue = on-track mutation

Supplementary References

1 Lee, J. H. *et al.* Vaccine genetics of IGHV1-2 VRC01-class broadly neutralizing antibody precursor naïve human B cells. *NPJ Vaccines* **6**, 113 (2021). https://doi.org:10.1038/s41541-021-00376-7

2 Jardine, J. G. *et al.* Minimally Mutated HIV-1 Broadly Neutralizing Antibodies to Guide Reductionist Vaccine Design. *PLoS Pathog* **12**, e1005815 (2016). https://doi.org:10.1371/journal.ppat.1005815
